# Supplementary material for: Chirality-induced avalanche magnetization of magnetite by an RNA precursor
Source: Nat Commun. 2023 Oct 10;14:6351. doi: 10.1038/s41467-023-42130-8 (PMC10564924; doi:10.1038/s41467-023-42130-8)
Supplement: Supplementary file 1 — Supplementary Information [file 41467_2023_42130_MOESM1_ESM.pdf]

Supplementary information for

Chirality-induced avalanche magnetization of magnetite by an RNA precursor

S. Furkan Ozturk[1†], Deb Kumar Bhowmick[2], Yael Kapon[3], Yutao Sang[2], Anil Kumar[2], Yossi Paltiel[3], Ron Naaman[2], and Dimitar D. Sasselov[4]

[1]Department of Physics, Harvard University, Cambridge, MA 02138, USA
[2]Department of Chemical and Biological Physics, Weizmann Institute, Rehovot 76100, Israel
[3]Department of Applied Physics, The Hebrew University of Jerusalem, Jerusalem 91904, Israel
[4]Department of Astronomy, Harvard University, Cambridge, MA 02138, USA

[†]E-mail: sukrufurkanozturk@g.harvard.edu

Table of Contents

1. Materials 3

2. Synthesis and characterization of L- and D-RAO 3

3. Fabrication of nickel-gold surfaces 5

4. Fabrication of magnetite (Fe_3O_4) surfaces 5

5. Characterization of magnetite (Fe_3O_4) surfaces 7

5.1 AFM 7

5.2 FTIR 8

5.3 UV-VIS 9

5.4 SQUID 10

6. CD measurements 11

7. MOKE measurements 16

7.1 Spin-coating 17

7.2 Drop-casting 18

8. MOKE measurements with AHPAL molecules 22

9. mc-AFM measurements 23

10. SQUID measurements 26

11. Modified Ising model simulations 29

12. X-ray crystallographic data of RAO 31

12.1 X-ray crystallography measurements 31

12.2 D-RAO 31

12.3 L-RAO 36

1. Materials

Reagents and solvents were obtained from *Sigma-Aldrich, Thermo Fisher, Acros Organics, and Santa Cruz Biotechnology* and were used without further purification. A *Mettler Toledo* SevenEasy pH Meter S20 combined with a *ThermoFisher Scientific* Orion 8103BN Ross semi-micro pH electrode was used to measure and adjust the pH to the desired value. ^{1}H-Nuclear magnetic resonance (NMR) spectra were acquired using a *Bruker* Ultrashield 400 Plus or *Bruker* Ascend 400 operating at 400.13 MHz. The notations s, d, t, and m represent the multiplicities singlet, doublet, triplet, and multiplet signal, respectively. Chemical shifts (*δ*) are shown in ppm.

2. Synthesis and characterization of L- and D-RAO

Cyanamide (5 g, 0.12 mol, 2 eq) was added to a solution of enantiopure ribose (9 g, 0.06 mol, 1 eq) in aqueous ammonia (3.5 %, 10 mL). The resultant mixture was swirled at room temperature until all solid material was dissolved. After 30 min, the solution was maintained at 60ºC for a further 1 hour. The reaction mixture was then cooled to room temperature and methanol (10 mL) was added to promote crystallization. After 16 hours at 4 ºC, ribo-aminooxazoline (RAO) crystals were collected by filtration, washed with ice-cold methanol (20 mL) and dried under vacuum. ^{1}H-NMR spectrum of the crystals dissolved in deuterium oxide was subsequently acquired.

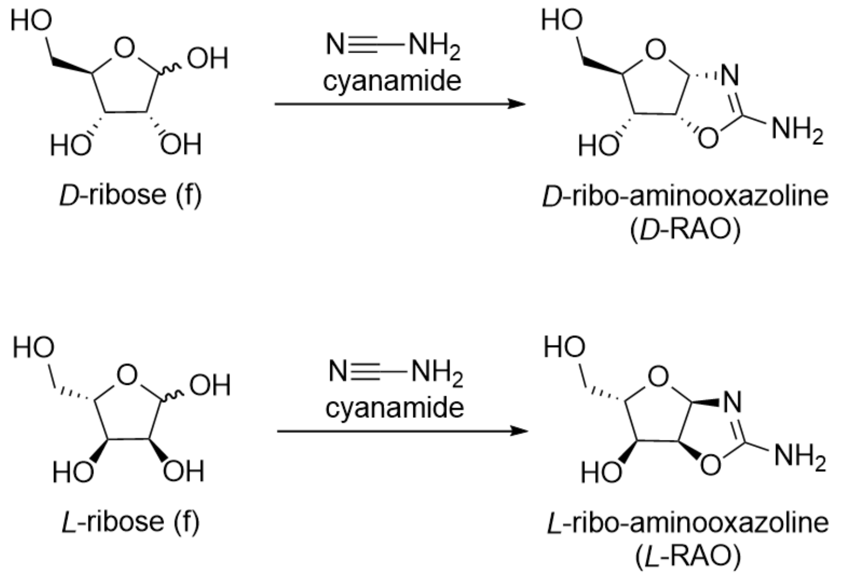

Suppl. Fig. 1. Enantiopure RAO was synthesized by the reaction of cyanamide with *D*- or *L*-ribose (furanose). After the incubation of ribose with cyanamide, RAO was crystallized from the reaction mixture and was obtained in high yields of up to 85%.

D-RAO (yield: 85 %): ^{1}H-NMR (400 MHz, Deuterium Oxide) δ 5.73 (d, *J* = 5.1 Hz, 1H), 4.91 (t, *J* = 5.3 Hz, 1H), 4.05 (dd, *J* = 9.6, 5.5 Hz, 1H), 3.86 (dd, *J* = 12.7, 2.4 Hz, 1H), 3.66 (dd, *J* = 12.7, 4.8 Hz, 1H), 3.54 (ddd, *J* = 9.6, 4.7, 2.4 Hz, 1H).

L-RAO (yield: 84 %): ^{1}H-NMR (400 MHz, Deuterium Oxide) δ 5.72 (d, *J* = 5.0 Hz, 1H), 4.90 (t, *J* = 5.3 Hz, 1H), 4.05 (dd, *J* = 9.5, 5.5 Hz, 1H), 3.85 (dd, *J* = 12.7, 2.4 Hz, 1H), 3.65 (dd, *J* = 12.7, 4.8 Hz, 1H), 3.54 (ddd, *J* = 9.5, 4.7, 2.4 Hz, 1H).

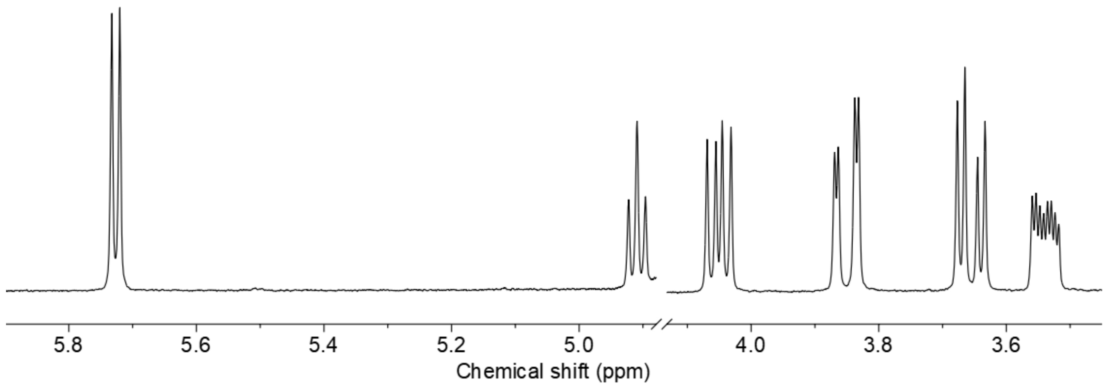

Suppl. Fig. 2. ^{1}H-NMR (400 MHz, Deuterium Oxide) spectrum of *D*-RAO.

Racemic RAO was obtained by mixing *D*- and *L*-RAO powders in equal amounts. Chiral properties of *D*-, *L*-, and *DL*-RAO were characterized by circular dichroism (CD) spectroscopy. For the CD measurements, RAO crystals were dissolved in pure water and after being diluted to about 70 μM CD spectra were acquired in quartz cuvettes with a path length of 10 mm, at 20°C. pH of the solution was not adjusted for the measurements and left as is.

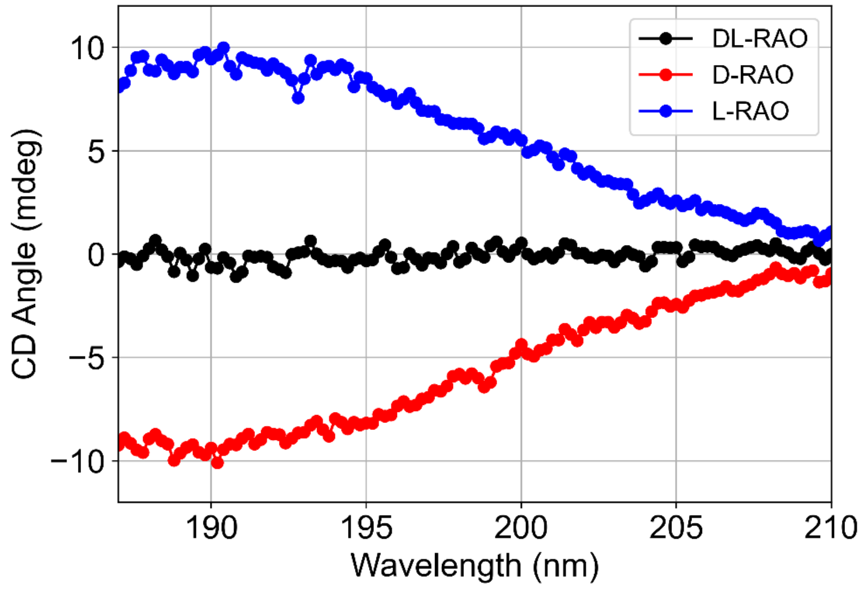

Suppl. Fig. 3. CD spectra of *D*- (red), *L*- (blue), and *DL*-RAO (black) in pure water (H_2O). A quartz cuvette with a path length of 10 mm is used and the spectra were taken for an RAO concentration of 70 [illegible]

3. Fabrication of nickel-gold surfaces

Ferromagnetic nickel-gold surfaces were fabricated for MOKE and mc-AFM measurements by evaporating an 8 nm titanium layer on silicon (100) wafers followed by a 30 nm layer of nickel and, finally, a 5 nm layer of gold at 3E-7 Torr. 100 mm wide and 0.5 mm thick Si (100) wafers (resistivity 0.001 to 0.005 ohm cm) by University Wafers were used. The layers were evaporated with an electron-beam evaporator by Odem Scientific Applications. The thin gold coating is used to prevent the oxidation of the ferromagnetic layer in air and in solution—preserving the magnetic and spin transport properties of the magnetic substrate. After the ferromagnetic samples were fabricated, they were kept in a vacuum desiccator to prevent oxidation and degradation due to the humidity.

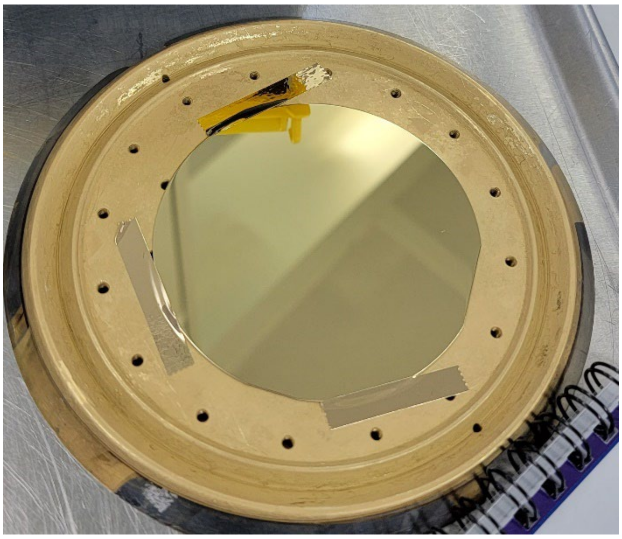

Suppl. Fig. 4. A nickel-gold substrate was fabricated by evaporating 8 nm titanium on a Si (100) wafer, followed by the deposition of 30 nm nickel and 5 nm gold layers under ultra-high vacuum. The magnetic substrate is obtained as a smooth layer with a shiny, metallic-gold appearance.

4. Fabrication of magnetite (Fe_3O_4) surfaces

Thin films of magnetite were fabricated using the procedure demonstrated by Jubb and Allen (2010) (*42*). For CD measurements transparent quartz substrates (1-mm-thick by Ted Pella, INC., PN: 26012); for SQUID measurements opaque silicon substrates (<100>, 100 mm by 0.5 mm by University Wafers) were used. First, iron films were deposited by the electron-beam evaporation of iron the substrate. For CD measurements 40 nm of iron and for SQUID measurements 100 nm of iron were deposited. For the evaporation, a thermal e-beam evaporator (SHARON EE-3 located at the Harvard Center for Nanoscale Systems) was used. The evaporation chamber was vacuumed down to 5E-6 Torr and for the iron evaporation a density of 7.86 g/cm³ and a z ratio of 0.349 were used at a deposition rate of 0.5 Å/s. After the deposition of iron, the substrates were placed in an oven (Binder Model FD 56 Laboratory Oven) at 175 ºC under ambient air and oxidized for 4 hours. Thus, the oxidization of iron (Fe) was promoted and an almost complete

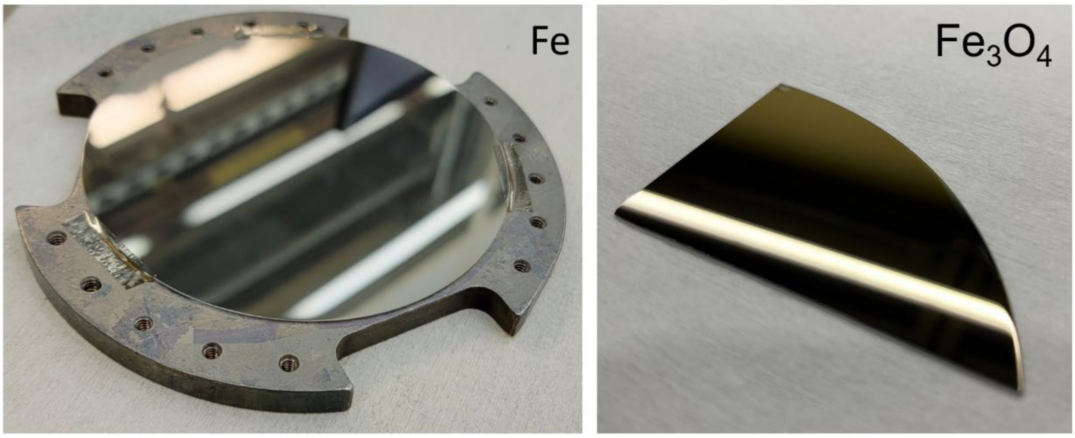

Suppl. Fig. 5. 100 nm thick iron (Fe) evaporated on a silicon substrate (0.5 mm thick) gives a metallic, shiny gray surface (left). After the oxidation of the film in air at 175ºC for 4 hours, iron oxidizes to a smooth film of about 200 nm magnetite, Fe_3O_4, with a green-yellow-black color (right).

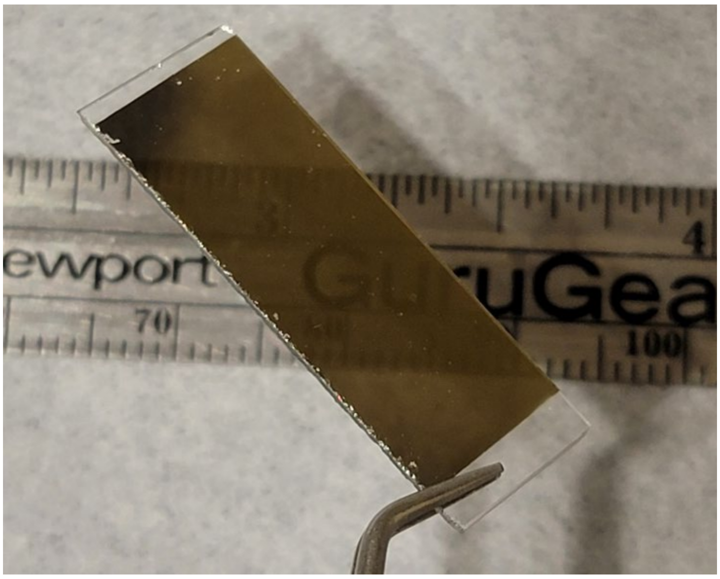

Suppl. Fig. 6. 80-nm-thick magnetite, Fe3O4, on a transparent 1-mm-thick quartz glass substrate is a semi-transparent magnetic surface suitable for solid-state CD measurements.

conversion to magnetite (Fe_3O_4) was achieved. We observed that the shiny gray iron film turned into a smooth layer of greenish-yellow, black magnetite film, as seen in Suppl. Fig. 5. Similarly, the 40-nm-film of Fe on the quartz glass substrate was oxidized to magnetite and a semi-transparent magnetic film was obtained. According to the cited procedure (*39*), 100 nm (40 nm) of iron should give around 200 nm (80 nm) of Fe_3O_4 after the oxidation due to oxygen incorporation into the iron lattice. After magnetite films were fabricated, they were kept in a vacuum desiccator to prevent the further oxidation to hematite and hydrous iron oxides.

5. Characterization of magnetite (Fe_3O_4) surfaces

5.1 AFM

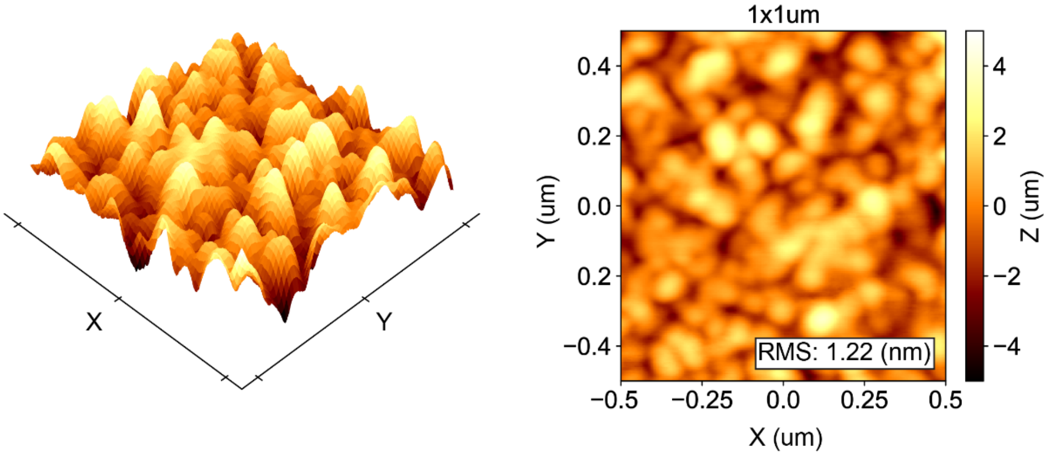

Suppl. Fig. 7: AFM topography image (3D image on the left, 2D top view on the right) of a magnetite film prepared from 100 nm iron deposited on a silicon wafer, with a slow deposition rate of 0.5 Å/s. Peak-to-peak surface roughness of 8.93 nm and an RMS roughness of 1.22 nm is measured for a randomly picked 1 x 1 µm area.

Surface properties of the magnetite films were characterized with a commercial AFM (Cypher S by Asylum Research) located at Harvard CNS facility. We used the AC mode (dynamic mode) and we imaged in air. We used a cantilever (Nanosensors Type SSS-NCH-10) with a nominal force constant of 42 N/m and a resonance frequency about 330 kHz. The cantilever oscillated at the auto-tuned resonance close to its frequency. The scan parameters were set at a scan rate of 1.00 Hz, an integral gain of 30.00, and a setpoint of 800.00 mV. The tip's amplitude and phase were monitored with a photodetector by using the tip deflection. We took scan ranges of 1.0 x 1.0 µm, 5.0 x 5.0 µm, and 10.0 x 10.0 µm and calculated the root-mean-square (RMS) roughness from the 3D topography data.

Scan Range (µm)	Peak-to-peak (nm)	RMS Roughness (nm)
1.0 x 1.0	8.93	1.22
5.0 x 5.0	11.42	1.41
10.0 x 10.0	10.82	1.30

Suppl. Table 1: Peak-to-peak variations and RMS surface roughness are shown for three different scan ranges, randomly picked on a magnetite surface.

5.2 FTIR

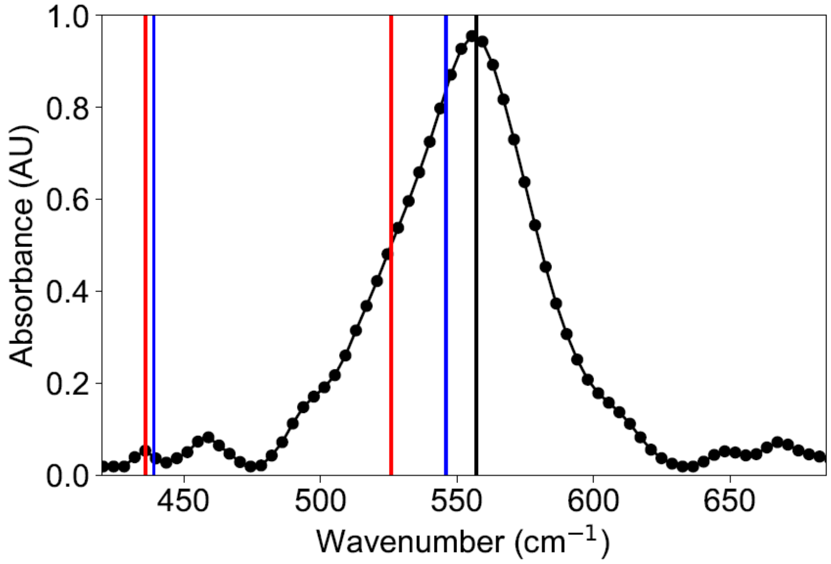

Suppl. Fig. 8: FTIR spectra of the 40 nm Fe_3O_4 films on 5-mm-thick KBr substrate. The prominent peak centered at 557 cm^{-1} (black line) is due to the magnetite absorption. Vertical blue and red lines are the expected locations of the maghemite and hematite respectively. FTIR spectra shows that iron films are selectively oxidized into magnetite.

The vibrational spectrum of the magnetite films was characterized by Fourier transform infrared (FTIR) spectroscopy. For FTIR measurements, we used a potassium bromide (KBr) window by ThorLabs (25 mm diameter, 5 mm thick, WG10255) due to its low absorption in the mid-IR range. We coated the KBr window with a 20 nm iron film using e-beam evaporation as described in Section 4. We used a commercial FTIR spectrometer by Bruker (Model: Invenio, 8000 cm^{-1} to 350 cm^{-1}). For the measurements, we used a resolution of 5 cm^{-1}, sample scan average of 100 scans, phase resolution of 16 in the Mertz phase correction mode. We used a MIR source and a KBr beam-splitter with a RT-DLaTGS detector. We took the measurements in the range of 700-350 cm^{-1}. We placed a KBr window into the background arm and the KBr/40 nm Fe_3O_4 sample into the sample arm of the spectrometer and used the background subtraction feature of the spectrometer. The measurement gave a prominent peak centered at 557 cm^{-1} (black vertical line in Suppl. Fig. 8) with a full width at half maximum of around 50 cm^{-1}, as shown in Suppl. Fig. 8. The central location of the peak agrees very well with the phonon mode splitting of magnetite, Fe_3O_4, as observed by Jubb and Allen (560 cm^{-1}) (*42*). We have not observed spectroscopic features of maghemite (439 and 546 cm^{-1}, blue vertical lines in Suppl. Fig. 8) and hematite (436 and 526 cm^{-1}, red vertical lines in Suppl. Fig. 8). We were not able to observe the magnetite mode at 350 cm^{-1} due to high background KBr absorption and we attribute the small ripples in the background to the residual water IR absorption. The absorption intensity we measure is not physical due to the reflection losses from the shiny magnetite film. FTIR spectra shows that iron films are selectively oxidized into magnetite and not into other iron oxides like maghemite (much less magnetic than magnetite) and hematite (not magnetic).

5.3 UV-VIS spectroscopy

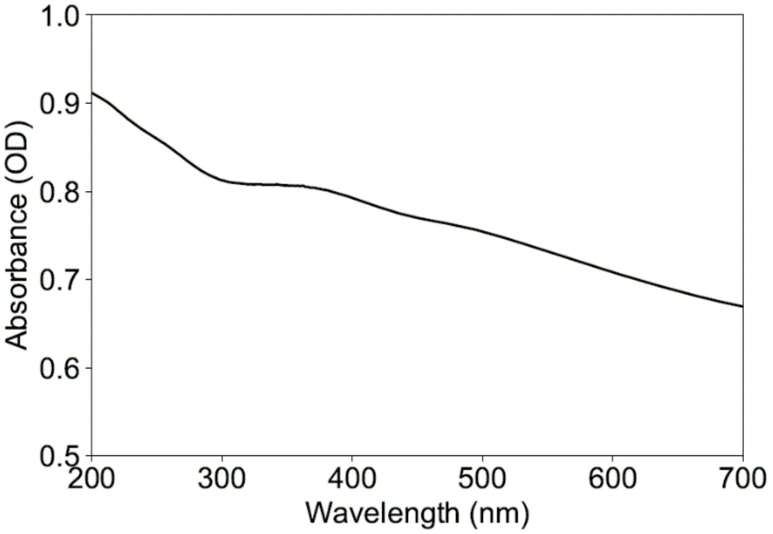

Suppl. Fig. 9: UV-Vis spectra of the 40 nm Fe_3O_4 films on 1-mm-thick fused silica substrate. Magnetite absorbs prominently in the visible range and it has a broad absorption feature slowly increasing into deep UV.

The optical spectrum of the magnetite films was characterized by ultraviolet–visible (UV-Vis) spectroscopy. For UV-Vis measurements, we used an uncoated, broadband UV fused silica window by ThorLabs (25.4 mm diameter, 1 mm thick, Part number: WG41050) due to its low absorption in the optical range. We coated the fused silica window with a 20 nm iron film and obtained a 40 nm magnetite film upon oxidation. We used a commercial UV-Vis spectrometer by Shimadzu (Model: UV-1900, 190 nm to 1100 nm). We took the measurements in the 190 – 700 nm range with a resolution of 2 nm, using the slow acquisition mode. We placed a fused silica window into the background arm and the fused silica/40 nm Fe_3O_4 sample into the sample arm of the spectrometer and obtained the background subtracted UV-Vis spectra. The measurement gave a broad absorption feature, slowly rising as the wavelength decreased as seen in Suppl. Fig. 9.

5.4 SQUID

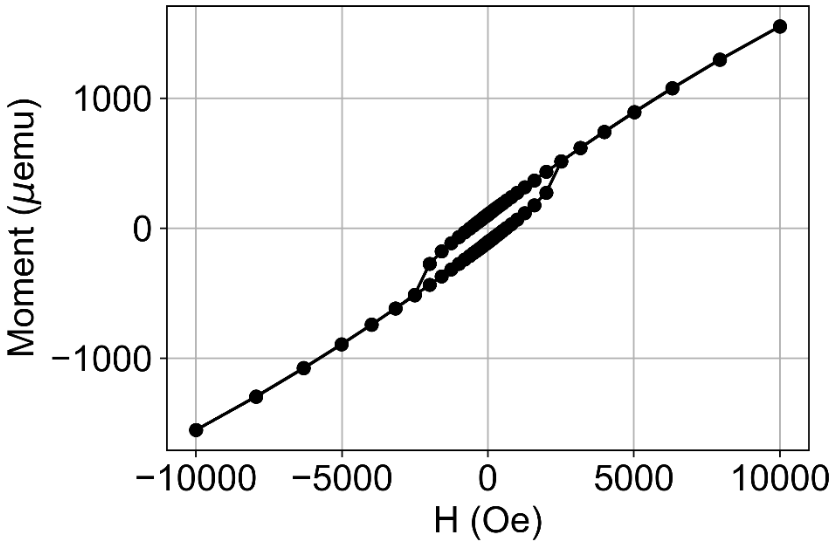

Suppl. Fig. 10: Hysteresis curve of a 200 nm magnetite sample on a 625 μm thick (4 mm by 4mm) Si wafer. The coercive field is measured to be around 600 Oe and as seen the sample does not saturate at 10 kOe field.

The magnetic properties of the magnetite samples were characterized by a superconducting quantum interference device (SQUID). We used the 200-nm-thick magnetite samples for the SQUID measurements on the silicon substrate. We took the measurements at room temperature by magnetizing the sample parallel to the surface normal. We separately measured the silicon substrate and subtracted its diamagnetic contribution. We measured the hysteresis curve of the sample with a magnetizing run from 0 Oe to 10 kOe, a first measurement from 10 kOe to -10 kOe, and a second one back to 10 kOe, ending with a demagnetizing run from 10 kOe to 0 Oe. We used a scan rate of 50 Oe/s. We measured the coercive field to be around 600 Oe, confirming the soft magnetic nature of magnetite. In addition, we observed that the sample does not saturate even at fields as high as 10 kOe, reaching a moment of above 1000 μemu.

6. CD measurements

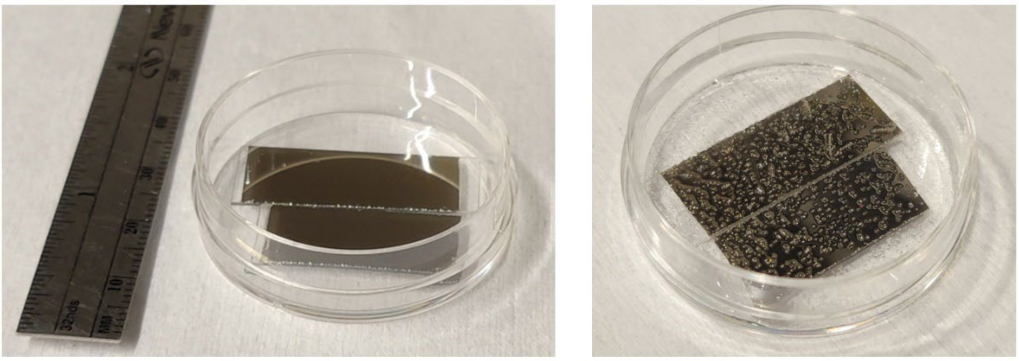

Suppl. Fig. 11: Photo on the left shows the magnetite surfaces on quartz placed inside a Petri dish before the crystallization. Photo on the right shows semi-transparent magnetite surfaces after a typical crystallization experiment with RAO crystals densely covering the magnetic surface.

Magnetization of the magnetic magnetite samples were measured by in-situ, solid state CD spectroscopy. For the CD experiments semi-transparent magnetite films on quartz were used. As shown in Suppl. Fig. 11, these magnetic samples were placed horizontally inside a 35 mm diameter Petri dish (Falcon Corning, Polystyrene, SN:351008, VWR#:25373-041) and the Petri dish was filled with a 2 mL aqueous solution of 75 mM enantiopure RAO such that the magnetic samples were fully covered with the solution. RAO was fully dissolved in 80ºC and it was made sure that no RAO crystal seeds were present in the solution prior crystallization. The setup was then overnight at room temperature for crystallization. After the crystallization is completed, supernatant was slowly pipetted out without disturbing the crystals on the surface and a magnetic surface densely covered with RAO crystals was obtained, as shown in the right panel of Suppl. Fig. 11. Then the surfaces were left open for a few hours for the crystals to dry and get firmly attached on the surface. After the surfaces were dried, their solid-state CD spectra were measured.

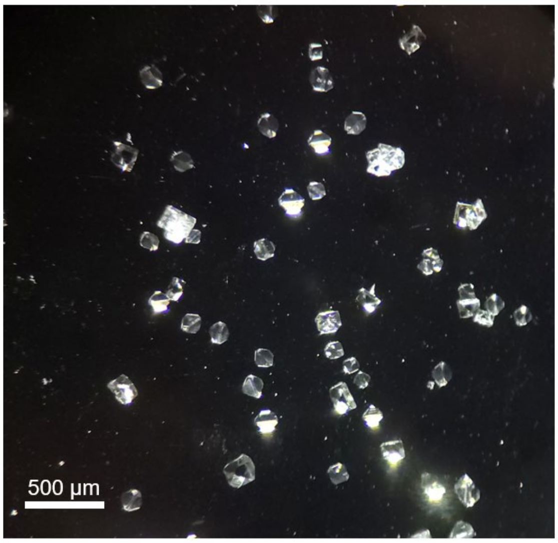

Suppl. Fig. 12: *D*-RAO crystals on the magnetite are shown after a typical crystallization experiment. Crystals are a few hundred microns in length, and they cover the magnetic surface.

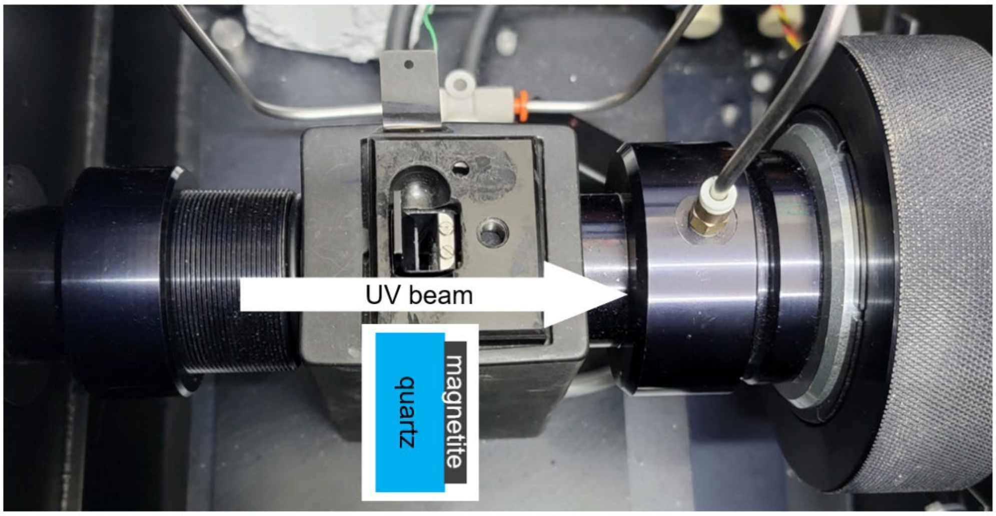

Suppl. Fig. 13: Magnetization of magnetite samples were measured by solid-state CD spectroscopy. Samples were placed upright and perpendicular to the beam direction, fully covering the beam aperture.

CD spectra were acquired by a Chirascan VX spectrometer (Applied Photophysics) with active temperature stabilization at 20ºC by a Quantum Northwest temperature controller. Before the measurements, CD spectrum of a quartz substrate was measured as a baseline—which is then subtracted from the signal. Substrates were held upright in a cuvette holder and placed perpendicular to the beam direction during the measurements as shown in Fig S13. It was made sure that the substrates fully cover the aperture of the spectrometer. Measurements were taken in the 210-600 nm wavelength range and the auto-subtraction feature was used to subtract the baseline. CD and UV/Vis absorption spectra were simultaneously measured and a step size and bandwidth of 1 nm, and a 1 second time per point were used for the acquisition.

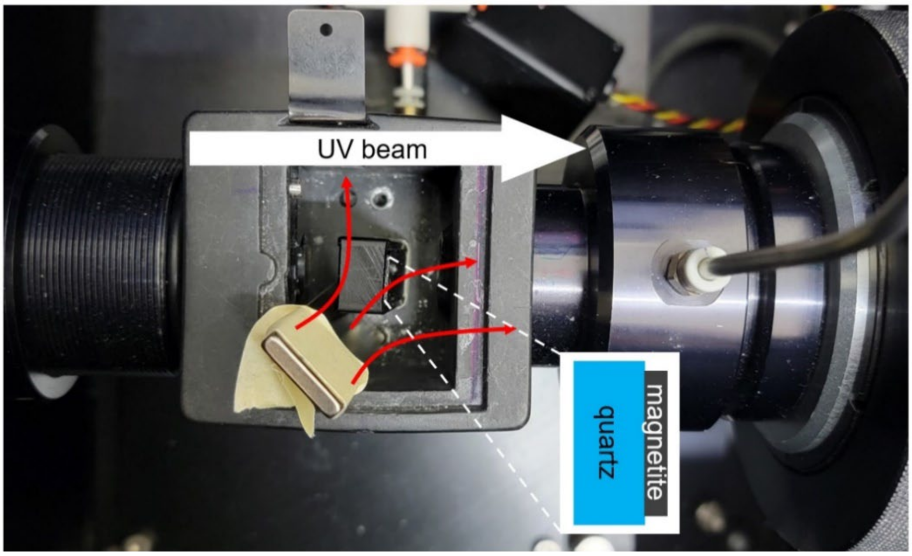

Suppl. Fig. 14: Magnetic magnetite samples were externally magnetized with a permanent magnet placed nearby the sample surface and the CD spectrum was acquired. The obtained CD spectra of the magnetized magnetite was used as a reference.

For the control experiment with externally magnetized magnetite, a neodymium magnet with a magnetization of 240 mT was placed at a 45 degrees angle and about 1 cm away from the substrate as shown in Fig S14. The out-of-plane magnetic field strength and the pole direction at the substrate surface was measured with a Hall probe (Extech MF100 AC/DC magnetic field meter) to be about 16 mT.

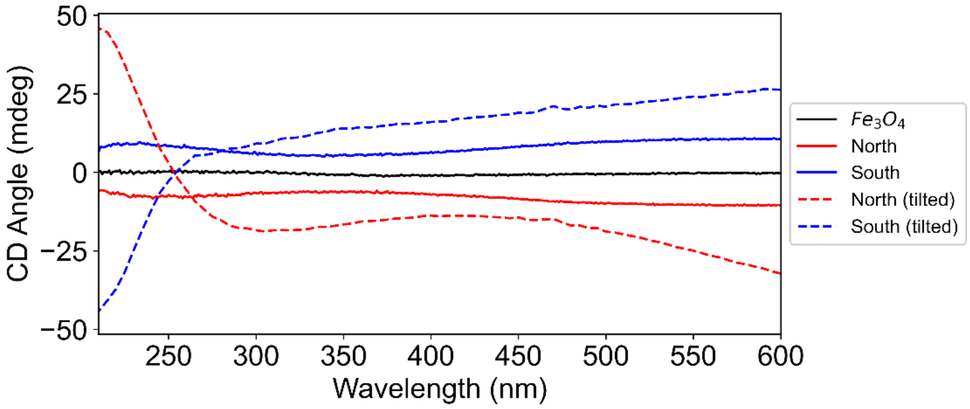

Suppl. Fig. 15: CD spectra of externally magnetized magnetite is dependent on the angle at which the surface is magnetized. Solid black line shows a non-magnetized magnetite while solid red and blue line show the CD signal of magnetized magnetite as it is mostly out-of-plane magnetized as placed in Suppl. Fig. 14. Dashed red and blue lines correspond to a magnetite surface tilted with respect to the UV beam such that the surface has nearly equal contributions of the in-plane and out-of-plane magnetizations.

It was found that spectral features of magnetized magnetite is dependent on the angle the surface was placed and when the surface is placed at an angle with respect to the beam we observed a zero-crossing of the CD signal at around 250 nm (Suppl. Fig. 14), although the features in the visible range above 350 nm were pretty much identical. This shows that in-plane and out-of-plane magnetized magnetite has different spectral features in the deep UV range yet a non-zero CD signal in the visible range (350-600 nm) is indicative of a net magnetization of magnetite.

Control experiments show that magnetite magnetized with the north (south) pole of the magnet has a broad positive (negative) CD signal in the visible range while the enantiopure RAO crystals absorb in the deep UV range. Therefore, the sign of the broad CD signal in the visible range is used as a proxy of the magnetization direction of magnetite. Enantiopure RAO (*D*- and *L*-separately) was crystallized on previously non-magnetized magnetite surfaces and the induced magnetization by the chiral molecules were probed. As a result, we observed that *D*-RAO (*L*-RAO) crystals magnetized the magnetite surface like the north pole (south pole) of a magnet with a positive (negative) CD signal. As shown in Suppl. Fig. 16-18, chirality-induced magnetization experiments have been repeated numerous times and the magnetization of the magnetite surfaces by the chiral RAO crystals was consistently observed.

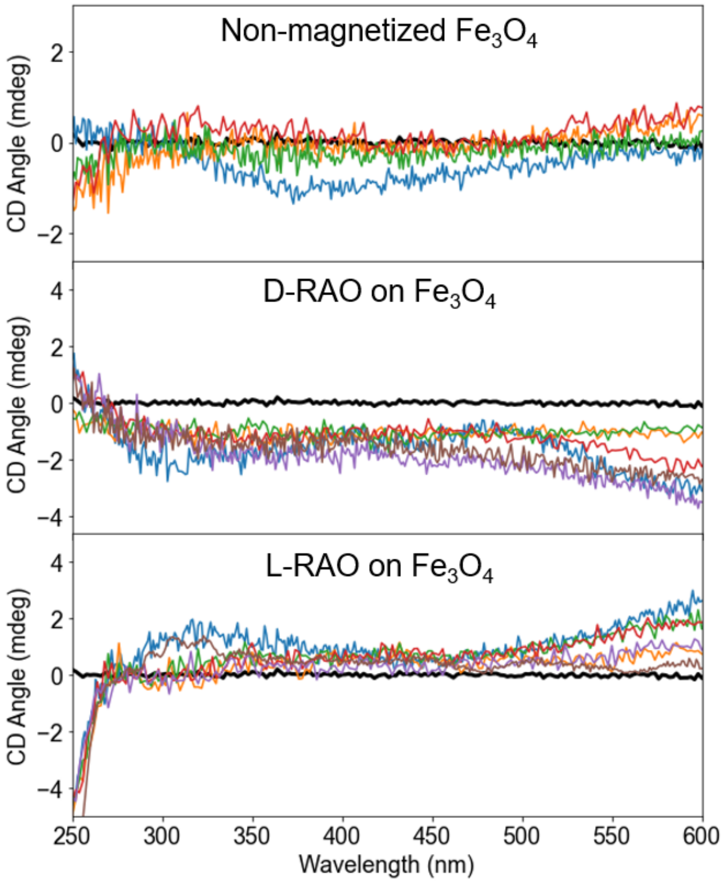

Suppl. Fig. 16: Chirality-induced magnetization of magnetite by enantiopure RAO crystals was repeated numerous times. Top Row: Magnetite surfaces with no net magnetization do not have a CD signal. Middle and Bottom Rows: Previously non-magnetized magnetite surfaces get magnetized by RAO crystals. *D*-RAO induces north-pole-like; *L*-RAO induces south-pole-like magnetization. Curves with different colors are repeated measurements and the black solid line shows the CD spectrum of a bare quartz surface as a baseline.

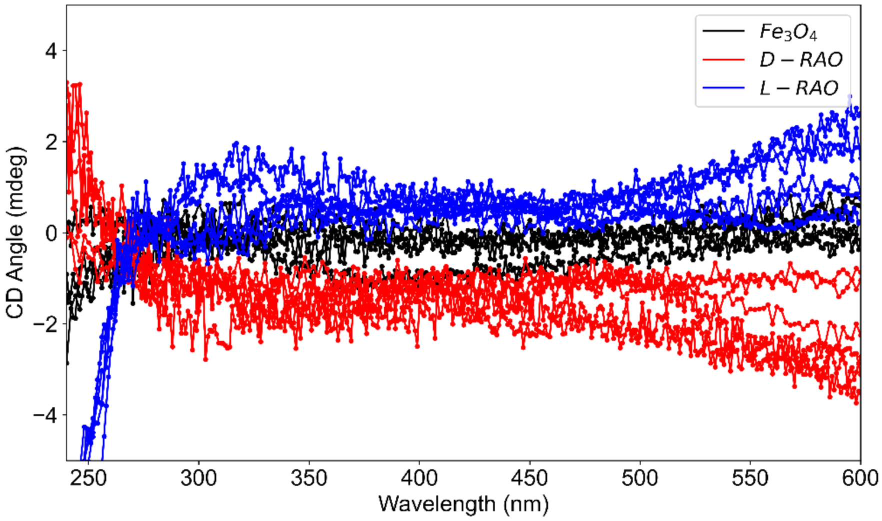

Suppl. Fig. 17: Chirality-induced magnetization of magnetite by RAO was consistently repeated in multiple experiments and repeated CD measurements are displayed together. Black curves show the CD measurements of magnetite with no net magnetization. Red and blue curves show the CD spectra of magnetite magnetized by *D*- and *L*-RAO crystals, respectively, in 6 different experiments each.

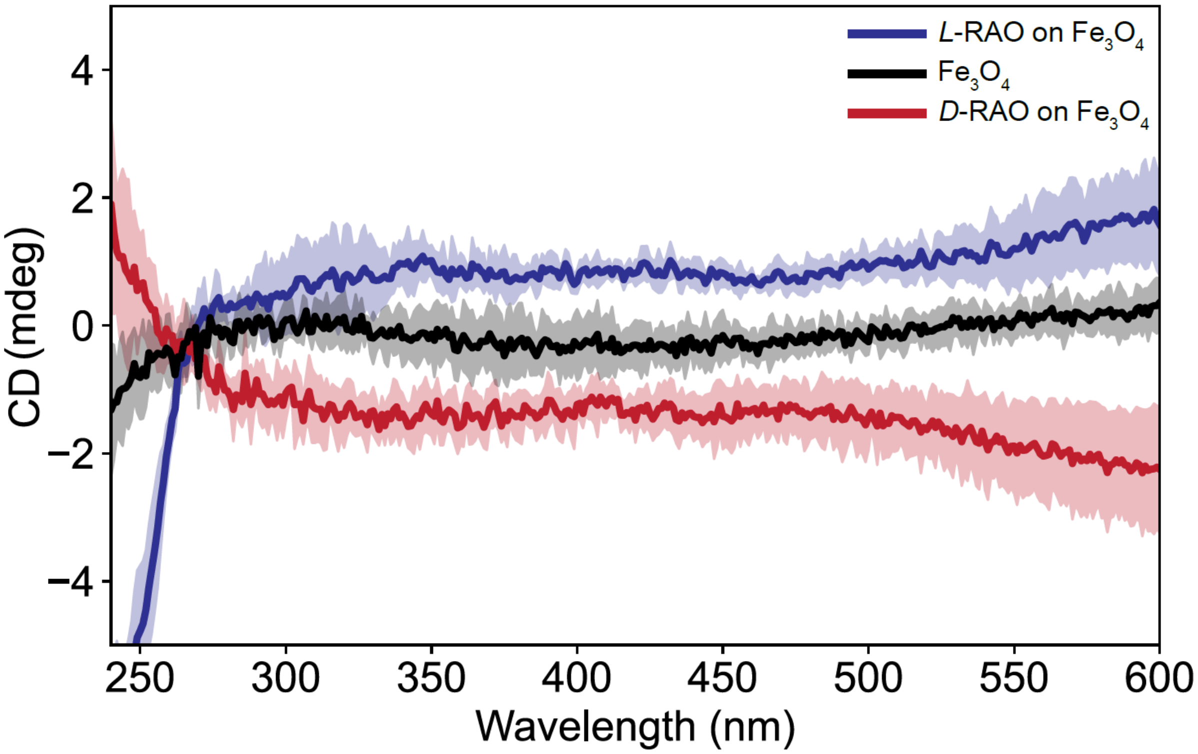

Suppl. Fig. 18: **Repeated CD measurements show consistent magnetization of Fe_3O_4 surfaces by RAO.** Chirality-induced magnetization of magnetite (Fe_3O_4) by RAO crystallization was consistently repeated in multiple experiments and the averaged CD spectra are displayed together. The black curve is the CD spectrum of magnetite with no net magnetization, averaged over 4 measurements. Red and blue curves are the CD spectra of magnetite magnetized by *D*- and *L*-RAO crystals, respectively, averaged over 6 measurements each. Shaded regions correspond to one standard deviation from the mean, for each averaged curve.

7. MOKE measurements

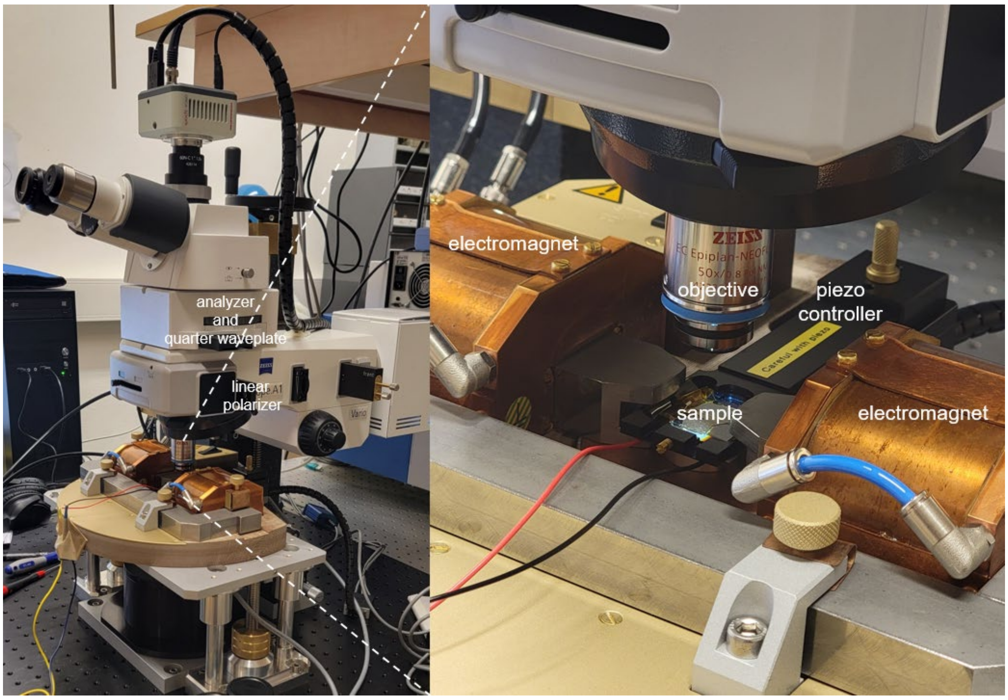

Suppl. Fig. 19: Magneto-optical Kerr effect microscope (MOKE) setup is displayed. The magnetic surface is imaged by a Zeiss objective and an in-plane magnetic field was generated by an electromagnet placed around the imaging plane of the microscope, as shown on the right photo. During the measurements samples were actively stabilized by a piezo controller.

MOKE measurements were taken by a commercial Evico Magnetics GmbH magneto-optical Kerr microscope equipped with an electromagnet and a piezo controller for mechanical stabilization, as seen in Suppl. Fig. 19.

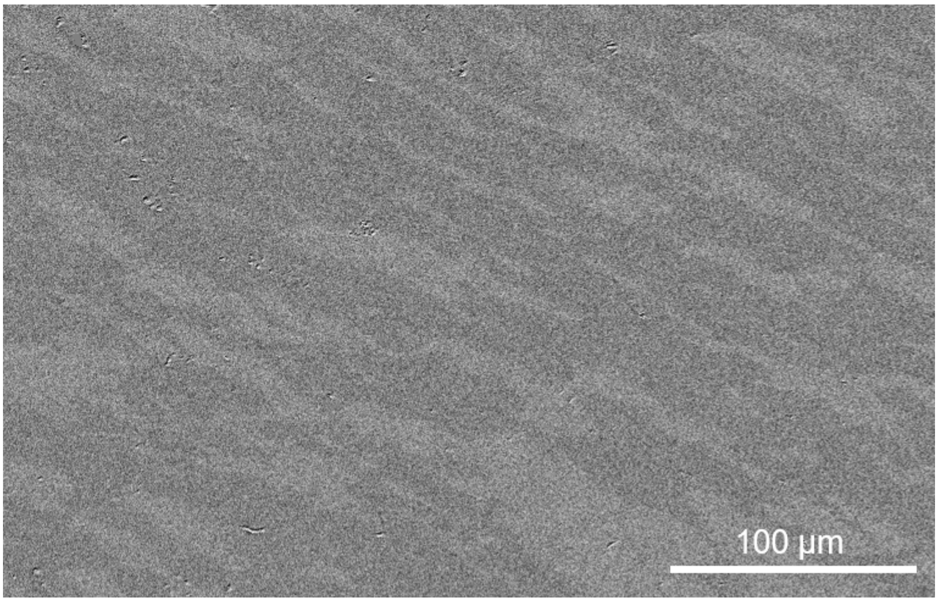

Suppl. Fig. 20: In-plane magnetic domains of the Ti/Ni/Au (8/30/5 nm) sample used for the MOKE measurements, in its demagnetized state. The domain size of the sample is measured to be around 10 microns.

For the MOKE measurements, ferromagnetic Ti/Ni/Au (8/30/5 nm) samples were used due to their domain size and their in-plane domains were imaged in the longitudinal configuration. In-

plane domains of the samples were imaged (Suppl. Fig. 20) in the demagnetized state, and they found to be suitable for the chirality-induced magnetization measurements: 1) they were large enough to be resolved by the optical microscope, 2) but they were not too large that they could still be flipped by chiral RAO crystals.

Magnetic samples with molecules (chiral and achiral) were prepared by two methods: spin-coating and drop-casting.

7.1 Spin-coating

A spin coater (WS series) by Laurel Technologies was used to form RAO crystals on the magnetic surface. In order to create a thin line of crystals on the surface a tape was used to cover a part of the surface and create a barrier nearby which the molecular solution is crystallized as thin white line, as shown in Fig S21. On the exposed side of the magnetic surface, a drop of 20 mM RAO dissolved in pure water was placed and the surface was spined in two cycles: 1) 500 RPM for 10 seconds, 2) 3500 RPM for 20 seconds (Suppl. Fig. 22).

As the surface is spined, due to the centrifugal force, the solution was spread out and smeared out along the tape and crystals formed as a thin layer between two areas free of chiral crystals. After spin coating, the tape was left on the surface and the samples were dried for a few hours. The tape was taken out before the MOKE measurements and the surface

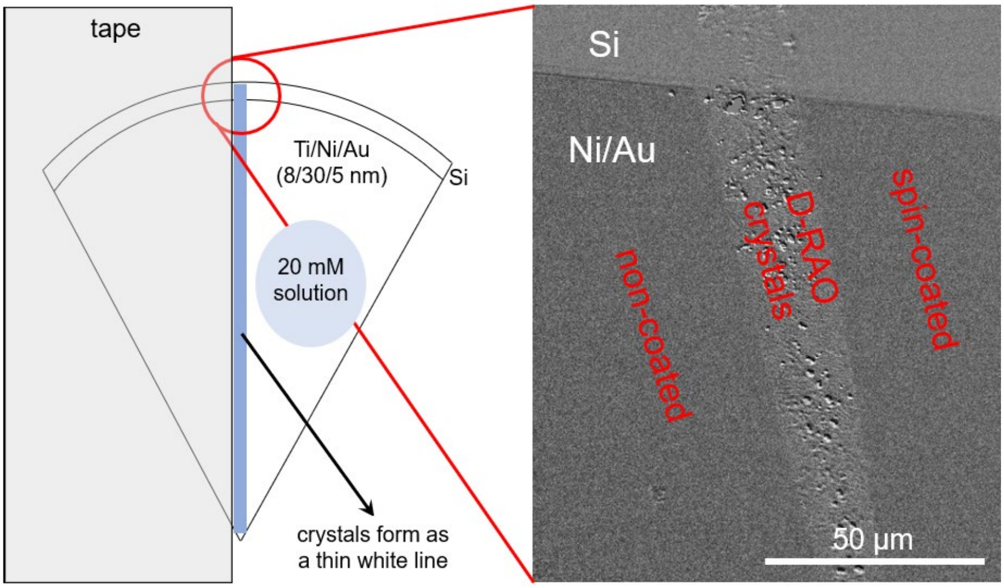

Suppl. Fig. 21: Enantiopure RAO crystals were formed as thin line by spin coating the magnetic Ni/Au surface. A tape was used to cover a side of the surface and molecular solution was dropped in the exposed area. Upon spinning the sample, the molecules crystallized at the boundary between the area covered by the tape and the exposed area.

was placed on the MOKE microscope such that the crystal line was perpendicular to in-plane magnetic field direction.

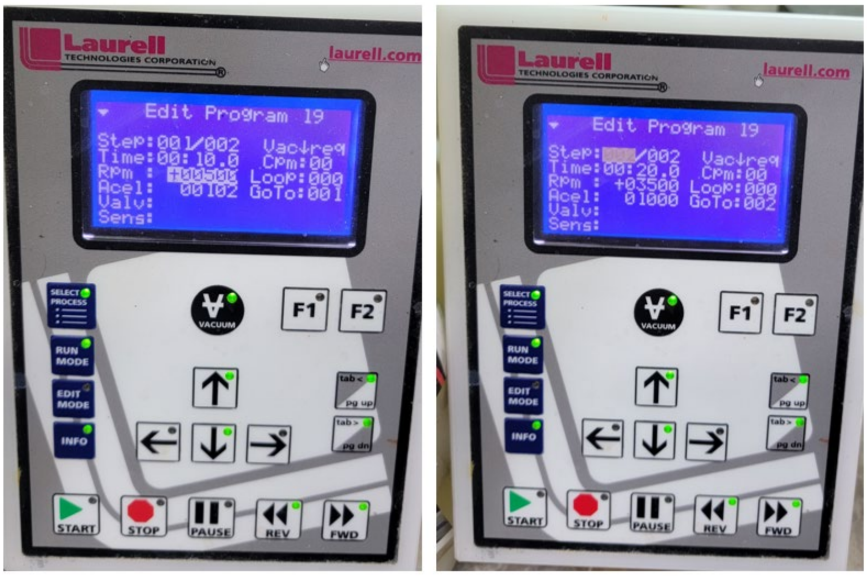

Suppl. Fig. 22: Spin coating parameters for MOKE and mc-AFM measurements for which 20 mM aqueous solutions of RAO were used.

7.2 Drop-casting

Second, drop-casting the solutions on the magnetic surface was used to create a molecular layer and form crystals on the surface, as a simpler method. In this method, we simply dropped a 5 µL solution of 20 mM *L*-, *D*-RAO or 20 mM NaCl or glycine on the Ni/Au surface. After the drop was placed on the clean surface, the sample was then placed in the fridge (-18°C) for two cycles of two hours, with a two-hour interval at room temperature. Finally, the sample was dried overnight at room temperature. As a result, we obtained areas of amorphous aggregation and areas of dense crystallization as seen in the optical images (Suppl. Fig. 23 and Suppl. Fig. 24).

As a control experiment, we formed enantiopure *D*-RAO crystals on a surface with magnetic (Ni/Au) and non-magnetic (Si) areas, using the spin-coating method. We then imaged the boundary of the magnetic and non-magnetic areas and observed the effect (chirality-induced magnetization) of chiral crystals only on the magnetic side of the surface (Suppl. Fig. 27). This measurement confirms that the observed effect is not an imaging artifact and is due to a physical magnetization contrast.

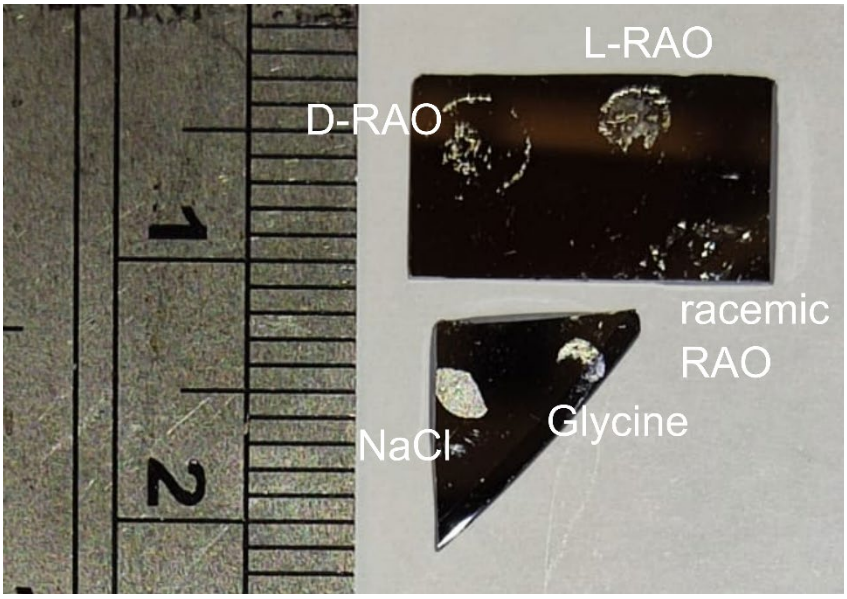

Suppl. Fig. 23: Crystals of *L*-, *D*-, *DL*-RAO and achiral NaCl and glycine on the ferromagnetic Ti(8nm)/Ni(30nm)/Au(5nm) substrate were formed by drop casting and subsequent freezing.

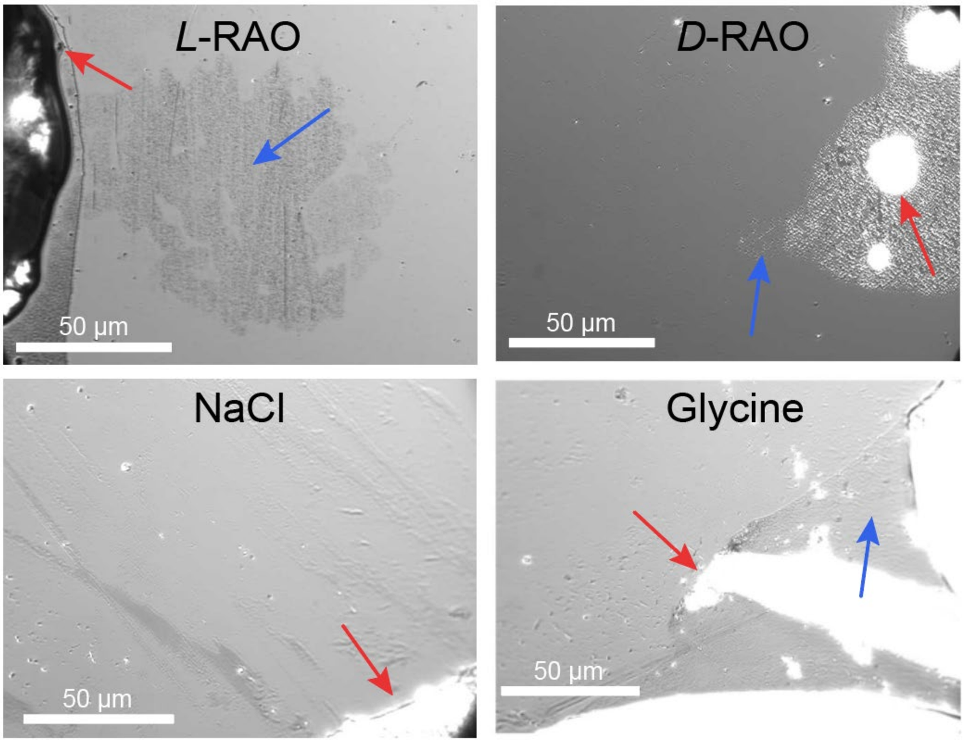

Suppl. Fig. 24: Optical images of *L*-, *D*-RAO and achiral NaCl and glycine crystals on the ferromagnetic Ti(8nm)/Ni(30nm)/Au(5nm) substrate. By repeated freezing of the droplets, we obtained areas of amorphous aggregation (red arrows) observed at the coffee ring of the dried droplet and areas of thinner crystalline coating (blue arrows). The thin coating allowed for domain imaging below the molecules. The domain images in Suppl. Fig. 25 correspond to the optical images.

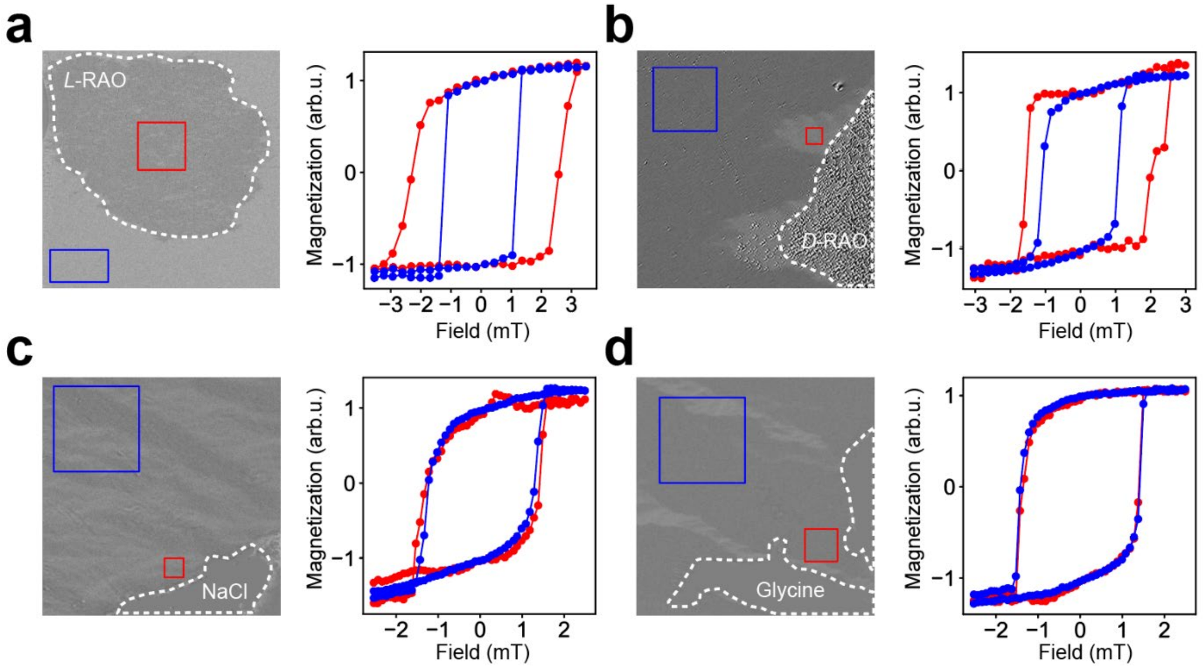

Suppl. Fig. 25: MOKE images and Kerr hysteresis curves of drop-casted compounds. Kerr hysteresis loops of magnetic surfaces were measured after L-RAO, D-RAO, NaCl and glycine crystals were formed on the surface. While chiral RAO crystals changed the magnetic behavior of the surface, achiral NaCl and glycine had no effect. For each hysteresis measurement, the blue (red) curve corresponds to the domains in the blue (red) square and white dashed lines show the drop-casted area, as confirmed by optical imaging. **a, b.** MOKE measurements of enantiopure L- and D-RAO exhibit a strong chirality-induced magnetization effect. For L-RAO we took the measurements before the crystals formed so we could directly image through the drop-casted area. With this we observed a strong increase in the coercivity by about 2 mT. For D-RAO we waited until the crystals form and imaged the nearby domains. We measured an increased coercivity of about 1 mT. The areas nearby (red) and far away from (blue) the chiral RAO crystals show different magnetic behavior as seen in the Kerr hysteresis curves. **c, d.** MOKE measurements with achiral compounds, sodium chloride (NaCl) and glycine, confirm that no magnetization is induced by the crystals of achiral compounds. The areas nearby (red) and far away from (blue) from the crystals show similar magnetic behavior, with nearly identical coercivities.

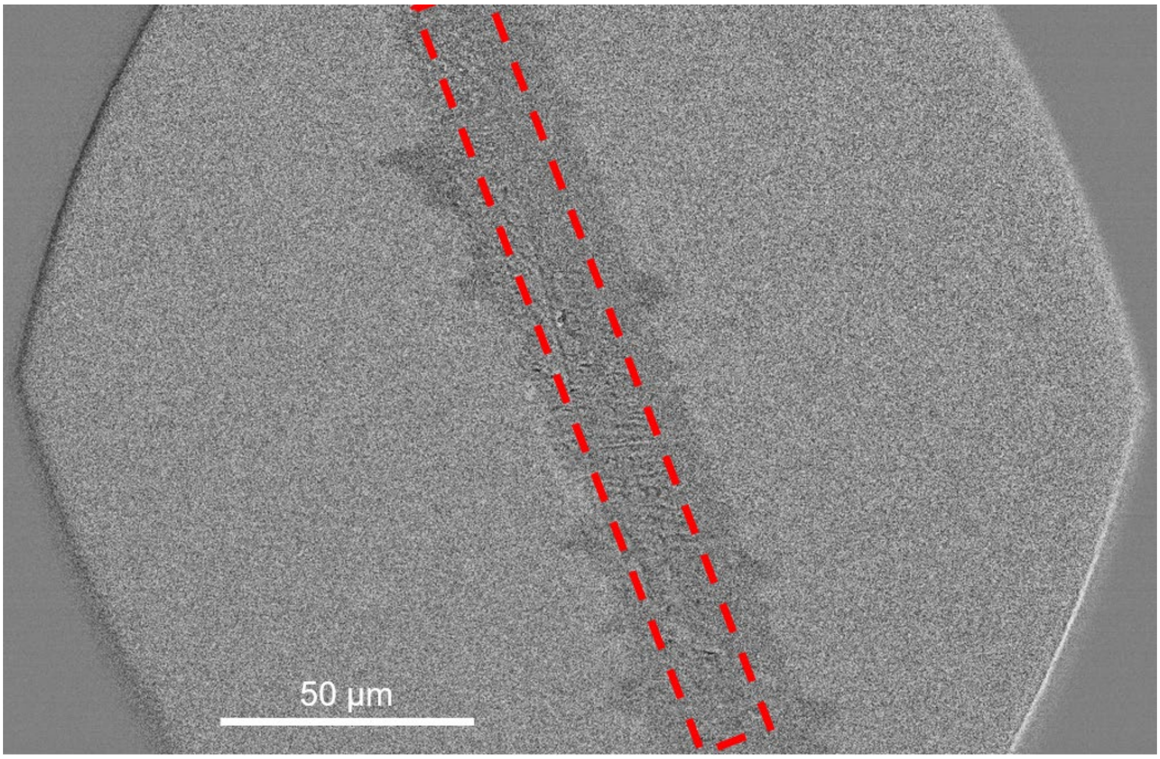

Suppl. Fig. 26: Full MOKE image of the Ni/Au surface magnetized by chiral *D*-RAO crystals. Dashed-red square shows the region where crystals form.

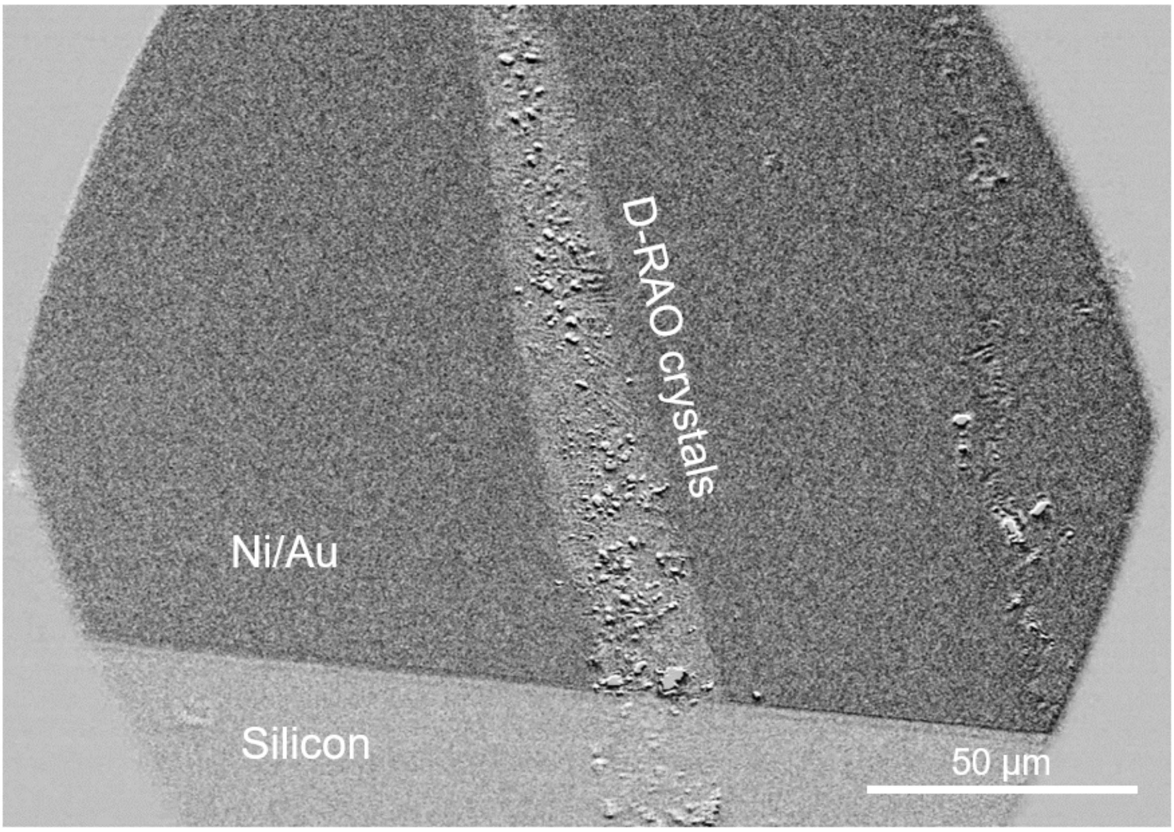

Suppl. Fig. 27: Chirality-induced magnetization by the spin-coated *D*-RAO crystals was observed only on the magnetic (Ni/Au) side of the surface. No image contrast was observed on the non-magnetic (Si) side, confirming that the observed effect is not an imaging artefact.

8. MOKE measurements with AHPAL molecules

To verify the generality of chirality-induced magnetization phenomenon beyond RAO, we studied the magnetization induced by α-helix polyalanine (AHPAL) molecules, which were adsorbed on magnetic nickel-gold surfaces (Suppl. Fig. 28).

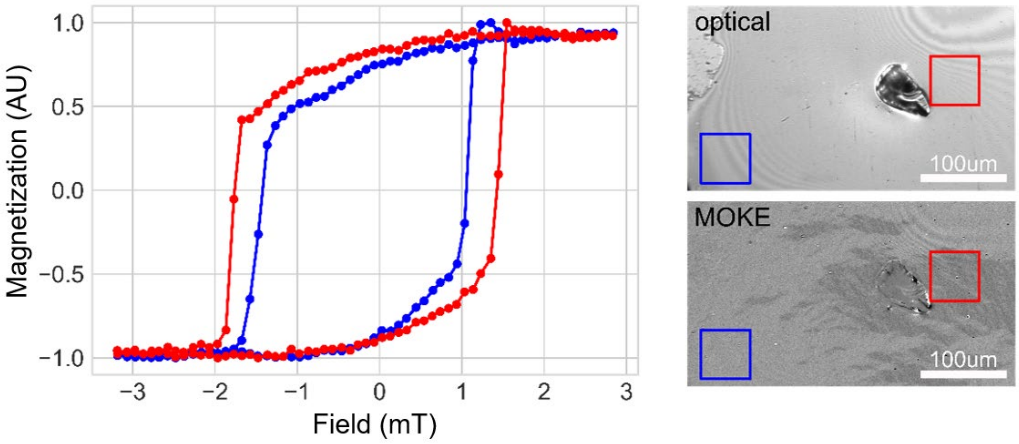

Suppl. Fig. 28: MOKE and optical images (right) and Kerr hysteresis curves (left) of the drop-casted L-alpha helix polyalanine molecules. Kerr hysteresis loops of magnetic surfaces were measured after L-alpha helix polyalanine aggregates were formed on the surface, as seen in the optical image (top right). Sufficiently away from L-alpha helix polyalanine chiral aggregates (blue box), the free domains have a coercive field of around 1 mT (blue line). However, an effective magnetic field with a spin-exchange origin interacts with the magnetic domains nearby the chiral molecules (red box). This local effective field provided by the chiral molecules translates into a higher coercive field of around 1.5 mT for the nearby magnetic domains (red line).

L-α-helix polyalanine [[H]-CAAAAKAAAAKAAAAKAAAAKAAAAKAAAAKAAAAK-[OH]] molecules (C stands for cysteine, A for alanine, and K for lysine) molecules were purchased and used in the experiments. A 1 mM solution of the molecules was prepared in ethanol and the solution was drop-casted on the magnetic substrate. A 1 μL drop was cast onto a Ti/Ni/Au (8/30/5 nm) surface. Then the sample was left in the air at room temperature until it completely dried. Visible aggregates were found by optical imaging in the region of the drop-cast and magnetic domain flipping by the chiral molecules was measured by MOKE imaging. The tiol end group of the α-helix polyalanine ensures a well-studied covalent bonding to the top gold layer of the ferromagnetic layer. Comparable systems were investigated in Ref. 17 and 18.

9. mc-AFM measurements

Samples for magnetic-conductive probe atomic force microscopy (mc-AFM) measurements were prepared by spin-coating aqueous solutions of enantiopure D- or L-RAO on Ti/Ni/Au (8/30/5 nm) surfaces. Magnetite surfaces could not be used for mc-AFM measurements due to the low conductivity of magnetite at room temperature. The concentration of the solution was picked such that enough current (> a few nA) could be passed in the -2V to +2V regime, where the instrument operates the best. Somewhere around 10-30 mM concentration was found to be workable, however, due to the thickness variations of the multi-layer coating an optimal spot was picked by local AFM measurements.

Magnetic field dependent current-voltage (I-V) characteristics of the prepared samples were determined using a multimodal scanning magnetic probe microscopy (SPM) system equipped with a Beetle Ambient AFM and an electromagnet with R9 electronic controller (RHK Technology). Voltage spectroscopy for the I-V measurements was performed by applying voltage ramps with a non-magnetic platinum tip (DPE-XSC11, MikroMasch with a spring constant of 3-5 N/m) in contact mode. During the mc-AFM measurements the magnetic substrate was kept magnetized with an external, out-of-plane magnetic field of 0.5 Tesla.

Numerous current-voltage measurements were taken for each enantiomer and magnetization direction, and they were averaged to obtain a clean I-V curve from which the spin-polarization is calculated. Two batches of I-V measurements were taken under identical preparation conditions, and they are displayed in Suppl. Fig. 29 (batch #1) and Suppl. Fig. 30 (batch #2). Curves with varying colors are single measurements for each batch and the red, thick line is the averaged I-V curve. The averaged I-V curves from batch #1 is shown in the main manuscript Fig. 5 due to its higher signal-to-noise ratio.

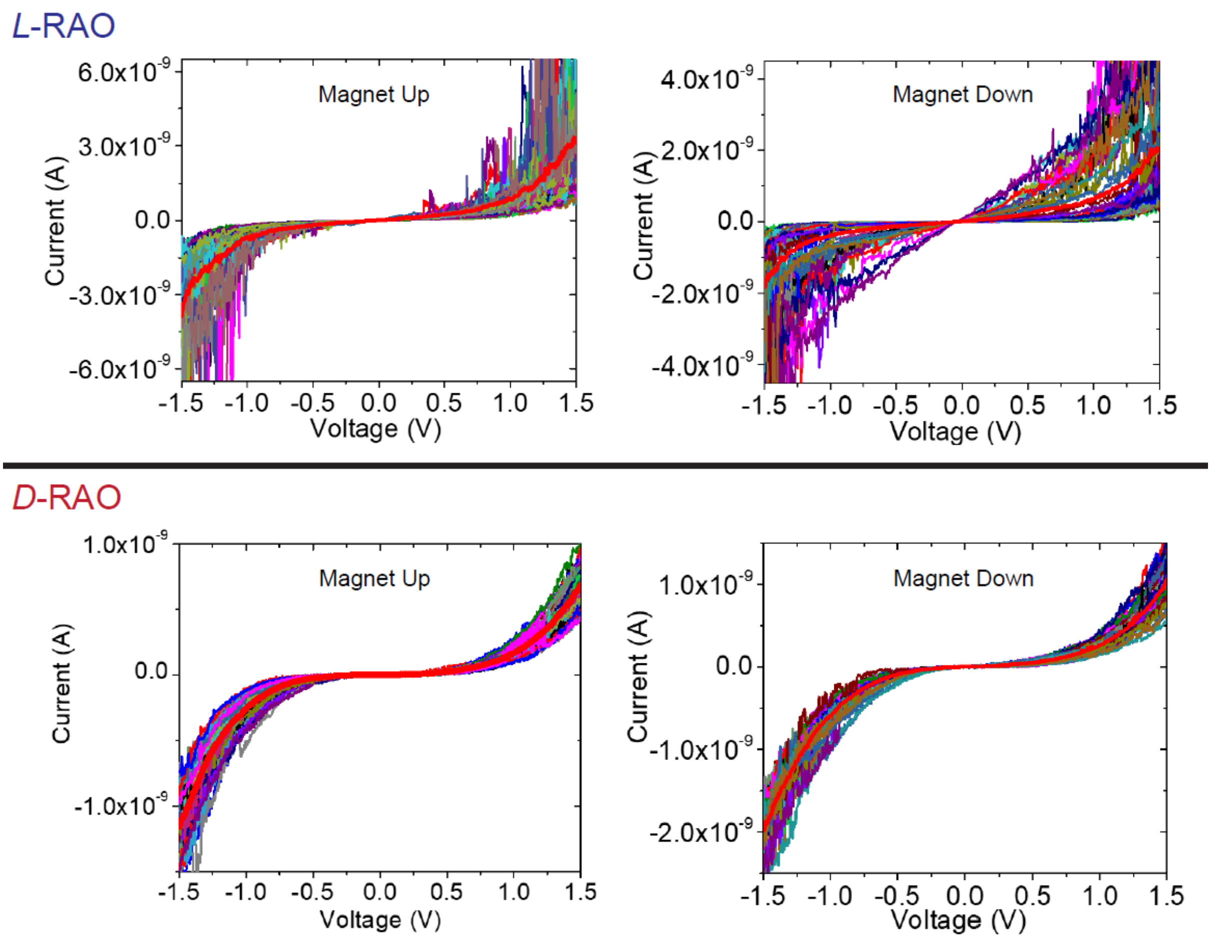

Suppl. Fig. 29: Magnetization dependent current-voltage (I-V) measurements (batch #1) for each enantiomer of RAO for up and down magnetization directions of Ni/Au. Colored curves represent a single I-V measurement, and the thick red line in each plot is the averaged curve over many single I-V measurements.

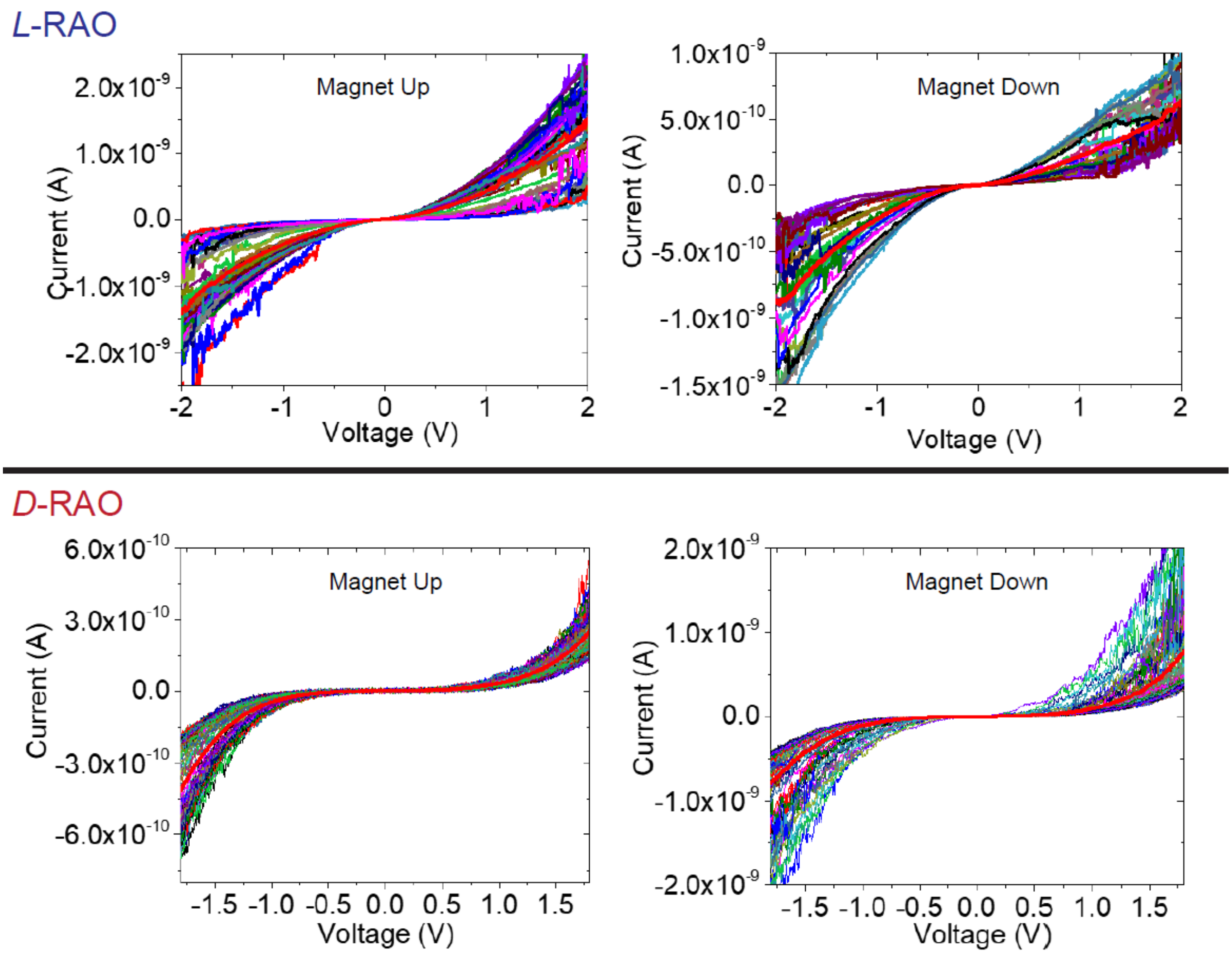

Suppl. Fig. 30: Magnetization dependent current-voltage (I-V) measurements (batch #2) for each enantiomer of RAO for up and down magnetization directions of Ni/Au. Colored curves represent a single I-V measurement, and the thick red line in each plot is the averaged curve over many single I-V measurements.

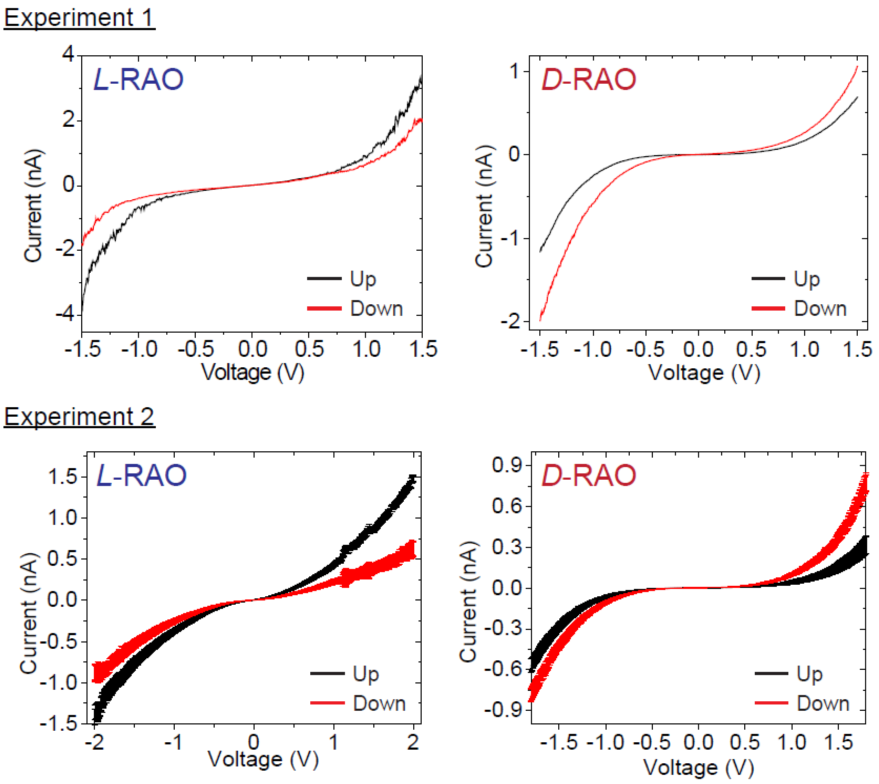

Suppl. Fig. 31: Averaged magnetization dependent current-voltage (I-V) curves for both batches. Experiment 1 is used to calculate the percent spin-polarization of RAO as and displayed the main manuscript, due to its higher signal-to-noise ratio.

10. SQUID measurements

SQUID measurements were taken to measure the induced magnetization of magnetite surfaces at zero field by D- and L-RAO crystals. For the measurements, 200 nm or 80-nm-thick magnetite surfaces formed on 0.5-mm-thick Si substrates cut to a square of 4 mm x 4 mm were used. On these surfaces enantiopure RAO was crystallized from its 75 mM aqueous solutions, as described in Section 6. With that magnetic samples densely coated by enantiopure RAO were obtained, as shown in Suppl. Fig. 32.

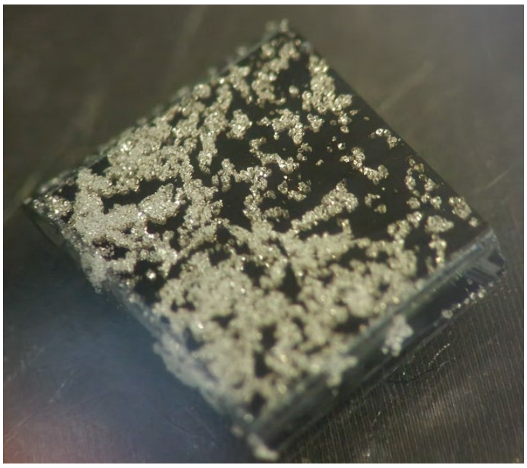

Suppl. Fig. 32: RAO was crystallized on magnetite samples (4 mm by 4 mm) for SQUID measurements.

Magnetic measurements of oxide films were performed using MPMS3 SQUID magnetometer (LOT-Quantum Design Inc.) by applying a vibrating sample mode. Induced magnetization of the samples was measured with the magnetic field parallel to the surface normal (out-of-plane). Samples were placed in a plastic straw. In order to center the sample position, a small magnetic field is applied and the so-called *lastscan* measurement was taken. Magnetization of the sample was measured at 300 K, while the magnetic field H was decreased and increased in the range of −1 kOe ≤ H ≤ +1 kOe. After each measurement, the substrate was soaked in water for 1.5 hours to thoroughly remove the crystals and then measured again under identical conditions. Therefore, we obtained the magnetization curves of the same substrate with and without crystals and corrected the magnetization curves with these baseline measurements.

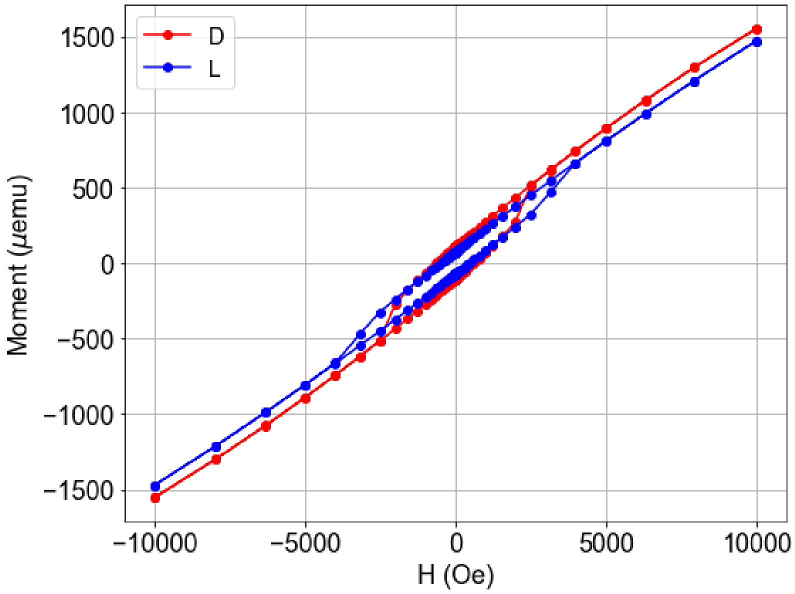

Suppl. Fig. 33: Magnetic hysteresis curves of magnetite surfaces with D- and L-RAO crystals.

First, the hysteresis curves of 200 nm magnetite surfaces with D- and L-RAO crystals were measured (Suppl. Fig. 33). We have not observed a significant difference between the curves. Although, with a thinner magnetite sample the induced surface magnetization can be amplified. In addition, as opposed to checking the difference between each enantiomer, the difference between the coercivities of a bare magnetite surface and the surface with chiral crystals (D or L) can be measured.

Next, we attempted to measure the induced magnetization by chiral crystals at low to zero external field—in order not to contaminate the authentic magnetization. However, despite our best efforts, we could not observe a repeatable difference between surfaces that are magnetized by D and L enantiomers. In most cases, we observed a difference as seen in Suppl. Fig. 34, however, the sign of the magnetization for each enantiomer was alternating from measurement to measurement. We attribute this inconsistency to the following reasons. First, due to diamagnetic contribution of RAO crystals, each magnetization measurement was contaminated in a non-controllable way. As it is not possible to subtract a common background due to the varying mass of RAO, each experiment had a different diamagnetic contribution to the magnetization. Second,

due to the vibrating nature of the measurements (VSM), crystals were disturbed, and a clean measurement was hard to take, unlike MOKE and CD measurements. Finally, because we had to apply an external magnetic field to center the magnetic sample, we contaminated the induced magnetization by crystals from the beginning. All things considered; we don't think SQUID is a suitable experimental tool to measure small surface magnetizations at zero field. It is better suited to measure larger bulk magnetizations of samples and their hysteresis behavior at larger external fields. MOKE microscopy is the most suitable experimental tool to measure induced magnetization at the surface without disturbing the magnetic sample.

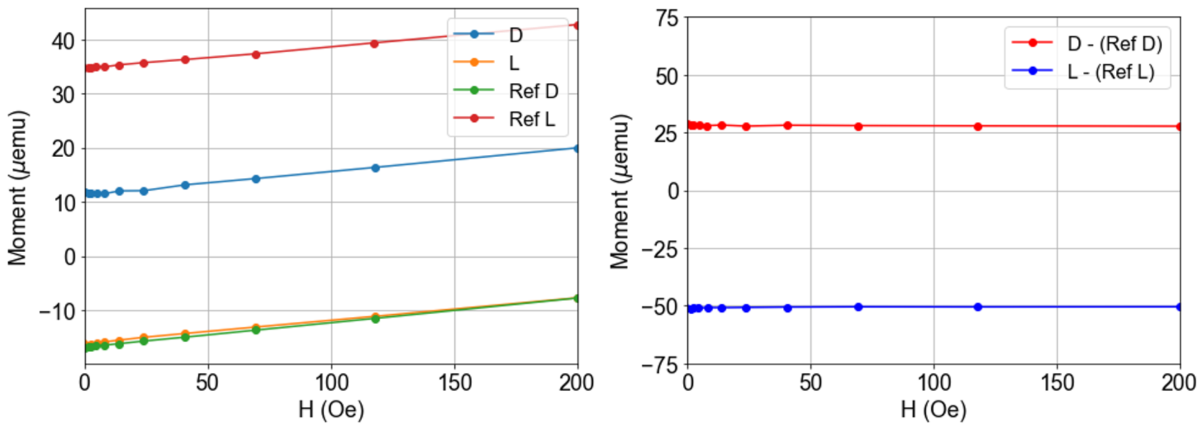

Suppl. Fig. 34: Low-field magnetization of magnetite by D- and L-RAO crystals is measured by a SQUID device. As seen on the left, after the crystals are removed a reference background measurement is taken for each surface and the subtracted curves for each enantiomer are displayed on the right. Although we observed a sign difference in the chirality-dependent (out-of-plane) magnetization of magnetite, we were not able to repeat the measurements consistently due to the diamagnetic contribution of the RAO crystals.

11. Modified Ising model simulations

A Monte-Carlo based simulation of the 2D Ising model was made to observe the avalanche magnetization by chiral crystals. The standard Ising model was modified to include the effect of chiral molecules at a basic level. Chiral molecules were modeled as static spins, σ_i, interacting with the ferromagnetic surface, S_i, due to the spin-exchange interaction, J_c.

$$H = -h_0 \sum_i S_i - J \sum_{\langle ij \rangle} S_i S_j - J_c \sum_{\langle ik \rangle} S_i \sigma_k$$

The evolution of the ferromagnetic spins was computed using the Metropolis-Hastings algorithm, using the following logic:

1. Create a random spin configuration: S(x, y)
2. Pick a random site: [i,j]
3. Calculate the energy of this site: H(i,j)
4. Decide to flip the spin or not:

   if H(i,j) < 0:

   Flip the spin

   else if random number(0,1) < P(H(i,j)): (where P is the Boltzmann probability)

   Flip the spin

   else:

   Do not flip the spin
5. Update the spin configuration and iterate

With this logic, first, the domain formation in the standard two-dimensional square lattice Ising model was simulated as shown in Suppl. Fig. 35 at a temperature below the critical temperature.

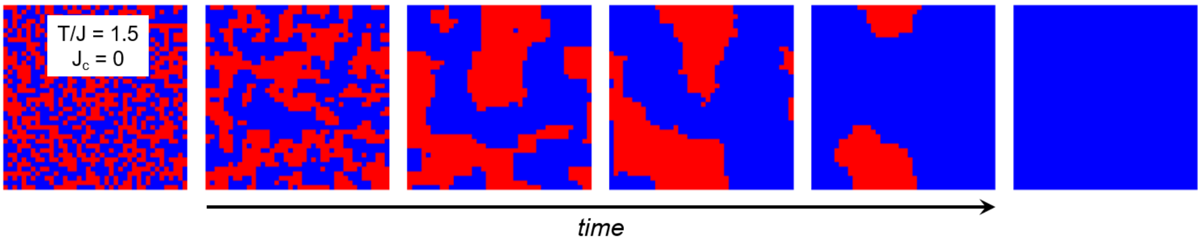

Suppl. Fig. 35: Magnetic domain formation was simulated by two-dimensional square lattice Ising model using a Monte-Carlo based simulation. The first frame on the left is the initial, random configuration of the spins (40 by 40 sites) where a red (blue) square indicates a spin-up (spin-down) lattice site. The second, third, fourth, fifth and sixth frames correspond to the spin configurations after 2, 10, 25, 100, and 500 iterations, respectively.

Next, the second-order phase transition at the critical temperature, T/J ~ 2.27, was simulated by calculating the temperature dependent magnetization of the system, as shown in Suppl. Fig. 36.

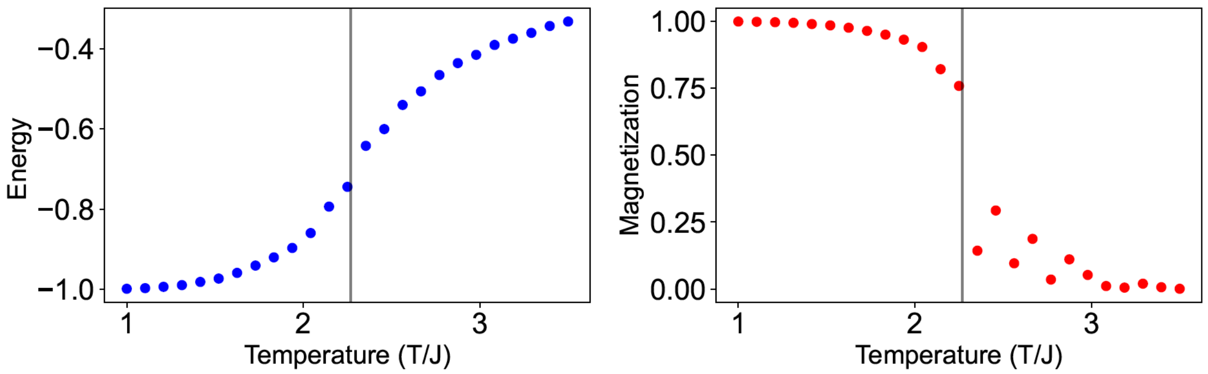

Suppl. Fig. 36: The second order phase transition in a standard two-dimensional square lattice Ising model was simulated and the drastic change of magnetization at the critical temperature was calculated as seen on the right.

Having confirmed that the simulation is working for the well-known, standard case of the Ising model, we simulated the effect of chiral crystals on a magnetic surface after the magnetic domains form, as shown in Suppl. Fig. 37.

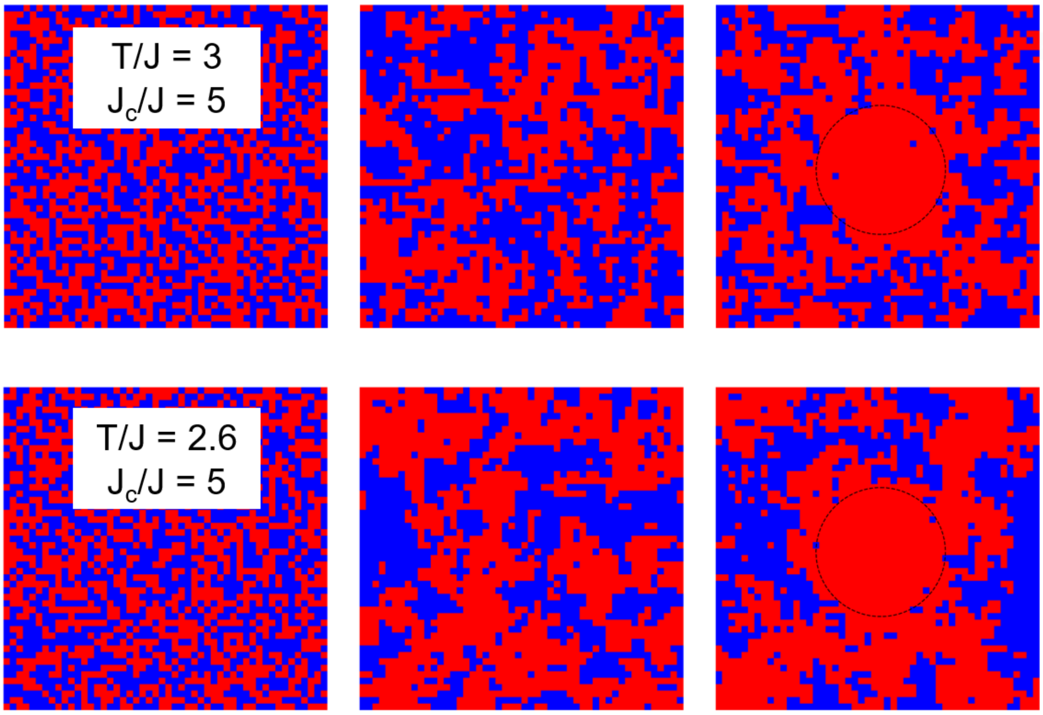

Suppl. Fig. 37: The effect of chiral crystals on a ferromagnetic surface due to the spin-exchange interaction ($J_c/J=5$) was simulated at two different temperatures. First, a random spin configuration (50 by 50, left column) is evolved and the magnetic domains form (central column). Next, chiral molecules are placed on the spin lattice on a region outlined by the dashed black circle. In the presence of chiral molecules, the domains flip and the induced magnetization spreads like an avalanche. The spread of magnetization is more wide-range at lower temperatures due to the formation of larger domains—if the spin-exchange energy of chiral molecules is kept constant.

12. X-ray crystallographic data of RAO

12.1 X-ray crystallography measurements

A crystal mounted on a diffractometer and data was collected at 100 K. The intensities of the reflections were collected by means of a Bruker D8 Venture diffractometer ($Cu_{K\alpha}$ radiation, λ=1.54178 Å), and equipped with an Oxford Cryosystems nitrogen flow apparatus. The collection method involved 1.0° scans in ω at -68°, -24°, 24°, 68°, and 113° in 2θ. Data integration down to 0.84 Å resolution was carried out. Non-hydrogen atoms were refined anisotropically, and hydrogen atoms were allowed to ride on the respective atoms. Crystal data as well as details of data collection and refinement are summarized in Tables S2, S5, geometric parameters are shown in Tables S3, S6 and hydrogen-bond parameters are listed in Tables S4, S7.

12.2 D-RAO

Experimental details

	D-RAO
Crystal data	
Chemical formula	$C_6H_{10}N_2O_4$
M_r	174.16
Crystal system, space group	Orthorhombic, $P2_12_12_1$
Temperature (K)	100
a, *b*, *c* (Å)	8.3674 (2), 8.5676 (2), 10.0411 (3)
V (Å^3)	719.83 (3)
Z	4
Radiation type	Cu $K\alpha$
μ (mm^{-1})	1.17
Crystal size (mm)	0.18 × 0.10 × 0.08
Data collection	
Diffractometer	Bruker D8 goniometer with Photon area detector

Absorption correction	Multi-scan<br>*SADABS*
T_{min}, T_{max}	0.681, 0.753
No. of measured, independent and observed [$I > 2\sigma(I)$] reflections	25337, 1270, 1261
R_{int}	0.030
$(\sin \theta/\lambda)_{max}$ (Å^{-1})	0.595
Refinement	
$R[F^2 > 2\sigma(F^2)]$, $wR(F^2)$, S	0.022, 0.058, 1.11
No. of reflections	1270
No. of parameters	126
H-atom treatment	H atoms treated by a mixture of independent and constrained refinement
$\Delta\rho_{max}$, $\Delta\rho_{min}$ (e Å^{-3})	0.21, -0.19
Absolute structure	Flack x determined using 506 quotients [(I+)-(I-)]/[(I+)+(I-)] (Parsons, Flack and Wagner, Acta Cryst. B69 (2013) 249-259).
Absolute structure parameter	0.04 (6)

Suppl. Table 2: Experimental details of the D-RAO crystal measurements.

Geometric parameters (Å, º)

O1—C1	1.412 (2)	N2—C4	1.454 (2)
O1—H1	0.87 (3)	C1—C2	1.525 (2)
O2—C3	1.358 (2)	C1—C5	1.526 (2)
O2—C2	1.4548 (19)	C1—H1C	1.0000
O3—C4	1.445 (2)	C2—C4	1.541 (3)
O3—C5	1.446 (2)	C2—H2	1.0000
O4—C6	1.425 (2)	C4—H4A	1.0000

O4—H4	0.86 (3)	C5—C6	1.511 (2)
N1—C3	1.336 (2)	C5—H5	1.0000
N1—H1A	0.89 (3)	C6—H6A	0.9900
N1—H1B	0.85 (3)	C6—H6B	0.9900
N2—C3	1.292 (2)		
C1—O1—H1	108.4 (16)	N2—C3—O2	118.18 (16)
C3—O2—C2	105.96 (13)	N1—C3—O2	113.86 (16)
C4—O3—C5	107.27 (13)	O3—C4—N2	111.75 (14)
C6—O4—H4	109.8 (18)	O3—C4—C2	105.62 (14)
C3—N1—H1A	118.5 (16)	N2—C4—C2	105.36 (13)
C3—N1—H1B	116.7 (16)	O3—C4—H4A	111.3
H1A—N1—H1B	120 (2)	N2—C4—H4A	111.3
C3—N2—C4	106.58 (15)	C2—C4—H4A	111.3
O1—C1—C2	112.15 (14)	O3—C5—C6	110.26 (14)
O1—C1—C5	113.34 (14)	O3—C5—C1	101.80 (13)
C2—C1—C5	102.43 (14)	C6—C5—C1	116.35 (15)
O1—C1—H1C	109.6	O3—C5—H5	109.4
C2—C1—H1C	109.6	C6—C5—H5	109.4
C5—C1—H1C	109.6	C1—C5—H5	109.4
O2—C2—C1	110.17 (13)	O4—C6—C5	112.96 (14)
O2—C2—C4	103.73 (13)	O4—C6—H6A	109.0
C1—C2—C4	104.23 (13)	C5—C6—H6A	109.0
O2—C2—H2	112.7	O4—C6—H6B	109.0
C1—C2—H2	112.7	C5—C6—H6B	109.0
C4—C2—H2	112.7	H6A—C6—H6B	107.8
N2—C3—N1	127.95 (17)		

C3—O2—C2—C1	113.85 (16)	C3—N2—C4—C2	4.20 (18)
C3—O2—C2—C4	2.79 (17)	O2—C2—C4—O3	114.17 (13)
O1—C1—C2—O2	36.69 (19)	C1—C2—C4—O3	-1.19 (17)
C5—C1—C2—O2	-85.16 (15)	O2—C2—C4—N2	-4.24 (17)
O1—C1—C2—C4	147.42 (14)	C1—C2—C4—N2	-119.59 (14)
C5—C1—C2—C4	25.57 (17)	C4—O3—C5—C6	166.13 (14)
C4—N2—C3—N1	176.76 (18)	C4—O3—C5—C1	42.05 (16)
C4—N2—C3—O2	-2.7 (2)	O1—C1—C5—O3	-162.05 (13)
C2—O2—C3—N2	-0.2 (2)	C2—C1—C5—O3	-41.01 (16)
C2—O2—C3—N1	-179.73 (14)	O1—C1—C5—C6	78.07 (19)
C5—O3—C4—N2	88.36 (16)	C2—C1—C5—C6	-160.89 (14)
C5—O3—C4—C2	-25.70 (16)	O3—C5—C6—O4	-59.37 (19)
C3—N2—C4—O3	-110.01 (15)	C1—C5—C6—O4	55.8 (2)

Suppl. Table 3: Geometric parameters of the D-RAO crystal.

Hydrogen-bond parameters

D—H⋯*A*	*D*—H (Å)	H⋯*A* (Å)	*D*⋯*A* (Å)	*D*—H⋯*A* (°)
O1—H1⋯O3^{i}	0.87 (3)	1.82 (3)	2.6759 (17)	168 (2)
O4—H4⋯N2^{i}	0.86 (3)	1.93 (3)	2.778 (2)	174 (3)
N1—H1B⋯O1^{ii}	0.85 (3)	2.28 (2)	2.862 (2)	126 (2)
N1—H1A⋯O4^{iii}	0.89 (3)	2.00 (3)	2.892 (2)	177 (2)

Suppl. Table 4: Hydrogen-bond parameters of the D-RAO crystal.

Symmetry code(s): (i) -*x*-3/2, -*y*-1, *z*+1/2; (ii) -*x*-3/2, -*y*-2, *z*-1/2; (iii) *x*, *y*-1, *z*.

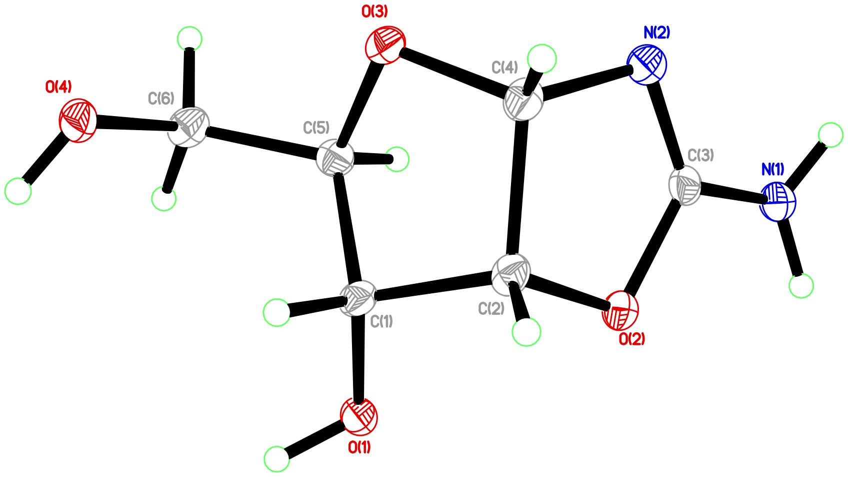

Suppl. Fig. 38. Perspective views showing 50% probability displacement for D-RAO.

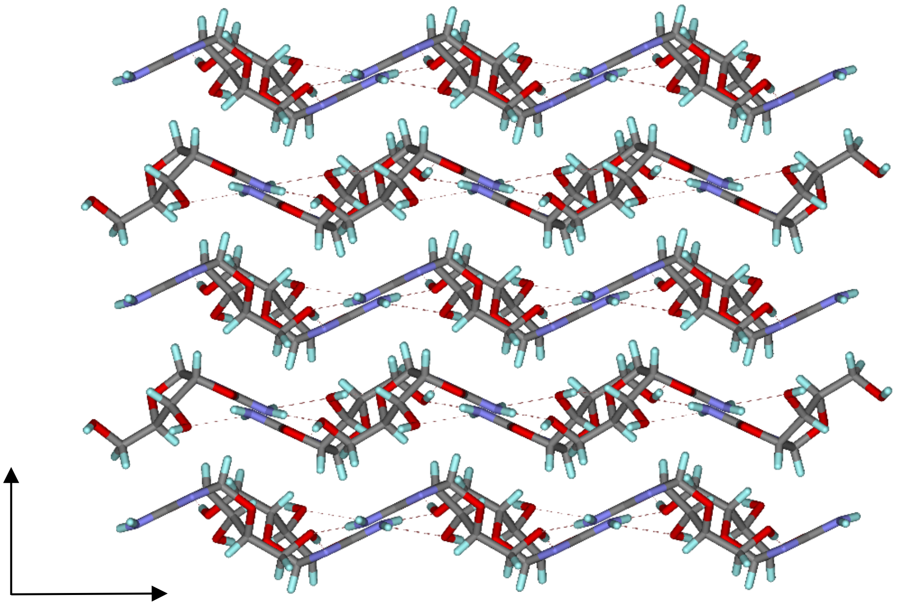

Suppl. Fig. 39. Three-dimensional supramolecular architecture viewed along the *c*-axis direction.

12.3 L-RAO

Experimental details

	L-RAO
Crystal data	
Chemical formula	$C_6H_{10}N_2O_4$
M_r	174.16
Crystal system, space group	Orthorhombic, $P2_12_12_1$
Temperature (K)	100
a, b, c (Å)	8.3646 (2), 8.5700 (2), 10.0383 (2)
V (Å^3)	719.59 (3)
Z	4
Radiation type	Cu $K\alpha$
μ (mm^{-1})	1.17
Crystal size (mm)	0.18 × 0.12 × 0.10
Data collection	
Diffractometer	Bruker D8 goniometer with Photon area detector
Absorption correction	Multi-scan<br>*SADABS*
T_{min}, T_{max}	0.757, 0.806
No. of measured, independent and observed [$I > 2\sigma(I)$] reflections	13526, 1264, 1250
R_{int}	0.032
$(\sin \theta/\lambda)_{max}$ (Å^{-1})	0.595
Refinement	
$R[F^2 > 2\sigma(F^2)]$, $wR(F^2)$, S	0.022, 0.057, 1.09
No. of reflections	1264
No. of parameters	126

H-atom treatment	H atoms treated by a mixture of independent and constrained refinement
$\Delta\rho_{max}$, $\Delta\rho_{min}$ (e Å^{-3})	0.19, -0.14
Absolute structure	Flack x determined using 499 quotients [(I+)-(I-)]/[(I+)+(I-)] (Parsons, Flack and Wagner, Acta Cryst. B69 (2013) 249-259).
Absolute structure parameter	-0.01 (6)

Suppl. Table 5: Experimental details of the L-RAO crystal measurements.

Geometric parameters (Å, º)

O1—C1	1.413 (2)	N2—C4	1.454 (2)
O1—H1	0.87 (2)	C1—C5	1.525 (2)
O2—C3	1.356 (2)	C1—C2	1.526 (2)
O2—C2	1.4554 (19)	C1—H1C	1.0000
O3—C4	1.444 (2)	C2—C4	1.540 (3)
O3—C5	1.446 (2)	C2—H2	1.0000
O4—C6	1.425 (2)	C4—H4A	1.0000
O4—H4	0.85 (3)	C5—C6	1.512 (2)
N1—C3	1.336 (2)	C5—H5	1.0000
N1—H1A	0.87 (3)	C6—H6A	0.9900
N1—H1B	0.86 (2)	C6—H6B	0.9900
N2—C3	1.292 (2)		
C1—O1—H1	110.1 (16)	N2—C3—O2	118.15 (16)
C3—O2—C2	105.99 (13)	N1—C3—O2	113.99 (16)
C4—O3—C5	107.26 (13)	O3—C4—N2	111.76 (14)
C6—O4—H4	110.1 (17)	O3—C4—C2	105.65 (14)
C3—N1—H1A	119.1 (16)	N2—C4—C2	105.36 (13)
C3—N1—H1B	117.5 (15)	O3—C4—H4A	111.3
H1A—N1—H1B	119 (2)	N2—C4—H4A	111.3

C3—N2—C4	106.59 (14)	C2—C4—H4A	111.3
O1—C1—C5	113.34 (14)	O3—C5—C6	110.23 (14)
O1—C1—C2	112.12 (14)	O3—C5—C1	101.77 (13)
C5—C1—C2	102.39 (14)	C6—C5—C1	116.29 (15)
O1—C1—H1C	109.6	O3—C5—H5	109.4
C5—C1—H1C	109.6	C6—C5—H5	109.4
C2—C1—H1C	109.6	C1—C5—H5	109.4
O2—C2—C1	110.18 (13)	O4—C6—C5	112.98 (14)
O2—C2—C4	103.71 (13)	O4—C6—H6A	109.0
C1—C2—C4	104.21 (13)	C5—C6—H6A	109.0
O2—C2—H2	112.7	O4—C6—H6B	109.0
C1—C2—H2	112.7	C5—C6—H6B	109.0
C4—C2—H2	112.7	H6A—C6—H6B	107.8
N2—C3—N1	127.85 (17)		
C3—O2—C2—C1	-113.90 (15)	C3—N2—C4—C2	-4.16 (19)
C3—O2—C2—C4	-2.87 (17)	O2—C2—C4—O3	-114.17 (14)
O1—C1—C2—O2	-36.71 (19)	C1—C2—C4—O3	1.17 (17)
C5—C1—C2—O2	85.10 (15)	O2—C2—C4—N2	4.26 (17)
O1—C1—C2—C4	-147.41 (14)	C1—C2—C4—N2	119.60 (14)
C5—C1—C2—C4	-25.60 (17)	C4—O3—C5—C6	-166.11 (14)
C4—N2—C3—N1	-176.68 (18)	C4—O3—C5—C1	-42.14 (16)
C4—N2—C3—O2	2.6 (2)	O1—C1—C5—O3	162.04 (13)
C2—O2—C3—N2	0.3 (2)	C2—C1—C5—O3	41.07 (16)
C2—O2—C3—N1	179.69 (14)	O1—C1—C5—C6	-78.17 (19)
C5—O3—C4—N2	-88.31 (16)	C2—C1—C5—C6	160.86 (14)
C5—O3—C4—C2	25.76 (16)	O3—C5—C6—O4	59.34 (19)
C3—N2—C4—O3	110.09 (16)	C1—C5—C6—O4	-55.8 (2)

Suppl. Table 6: Geometric parameters of the L-RAO crystal.

Hydrogen-bond parameters

D—H···*A*	*D*—H (Å)	H···*A* (Å)	*D*···*A* (Å)	*D*—H···*A* (°)
O1—H1···O3^{i}	0.87 (2)	1.83 (3)	2.6762 (17)	165 (2)
O4—H4···N2^{i}	0.85 (3)	1.94 (3)	2.7789 (19)	173 (2)
N1—H1B···O1^{ii}	0.86 (2)	2.27 (2)	2.8626 (19)	126.7 (18)
N1—H1A···O4^{iii}	0.87 (3)	2.03 (3)	2.893 (2)	177 (2)

Suppl. Table 7: Hydrogen-bond parameters of the L-RAO crystal.

Symmetry code(s): (i) -*x*+3/2, -*y*+1, *z*-1/2; (ii) -*x*+3/2, -*y*+2, *z*+1/2; (iii) *x*, *y*+1, *z*.

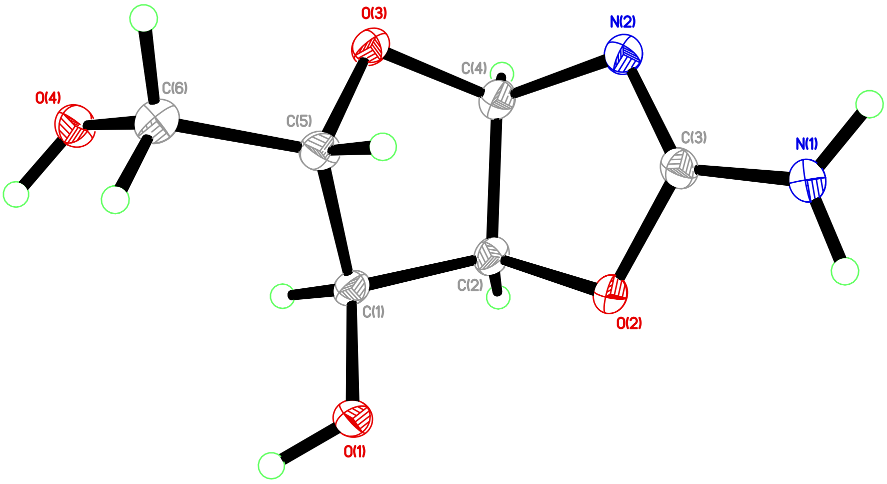

Suppl. Fig. 40*.* Perspective views showing 50% probability displacement for L-RAO.

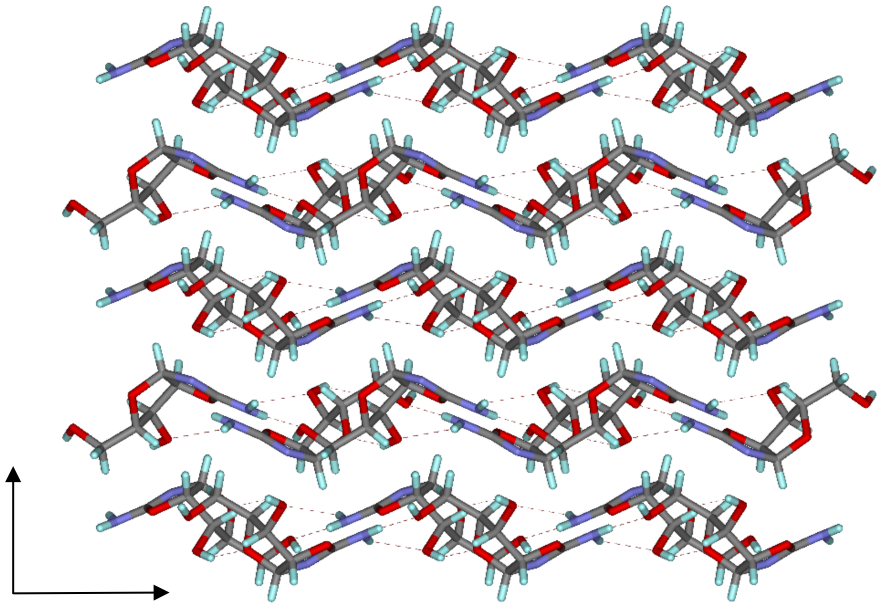

Suppl. Fig. 41. Three-dimensional supramolecular architecture viewed along the *c*-axis direction.
