# Supplementary material for: Chirality-induced avalanche magnetization of magnetite by an RNA precursor
Source: Nat Commun. 2023 Oct 10;14:6351. doi: 10.1038/s41467-023-42130-8 (PMC10564924; doi:10.1038/s41467-023-42130-8)
Supplement: Supplementary file 3 — Description of Additional Supplementary Files [file 41467_2023_42130_MOESM3_ESM.pdf]

Description of Additional Supplementary Files

File Name: Supplementary Movie 1
Description: Kerr hysteresis measurement of the Ni/Au surface with D-RAO crystals formed by spin-coating. Domains around the chiral crystals have a higher magnetic coercivity and they do not flip until a higher demagnetizing field is applied compared to the domains far from the chiral crystals.

File Name: Supplementary Movie 2
Description: Kerr hysteresis measurement of the Ni/Au surface with L-RAO crystals formed by drop-casting.

File Name: Supplementary Movie 3
Description: Kerr hysteresis measurement of the Ni/Au-Si surface with D-RAO crystals. Chirality-induced magnetization by chiral crystals was observed only on the magnetic (Ni/Au) side of the surface. No image contrast was observed on the non-magnetic (Si) side.

File Name: Supplementary Movie 4
Description: Kerr hysteresis measurement of the Ni/Au surface with achiral glycine crystals formed by dropcasting. Achiral crystals do not affect the magnetic properties of the surface.
